# Expression landscape of epigenetic genes in human hepatocellular carcinoma

**DOI:** 10.1007/s13105-025-01095-6

**Published:** 2025-06-12

**Authors:** Borja Castelló-Uribe, Amaya López-Pascual, Jasmin Elurbide, Elena Adán-Villaescusa, Emiliana Valbuena-Goiricelaya, Luz A. Martinez-Perez, Iker Uriarte, M. Ujúe Latasa, Bruno Sangro, María Arechederra, Carmen Berasain, Matías A. Avila, Maite G. Fernández-Barrena

**Affiliations:** 1 Hepatology Laboratory, Solid Tumors Program, CIMA, CCUN, University of Navarra, Pamplona, Spain; 2 Instituto de Investigaciones Sanitarias de Navarra IdiSNA, Pamplona, Spain; 3 CIBERehd, Instituto de Salud Carlos III, Madrid, Spain; 4 Liver Unit and HPB Oncology Area, CCUN, Navarra University Clinic, Pamplona, Spain; 5 Department of Health Sciences, Centro Universitario de los Altos (CUAltos), University of Guadalajara, Guadalajara, Mexico

**Keywords:** Hepatocellular carcinoma, Epigenetics, Gene expression, Oncofetal reprogramming, Immune landscape

## Abstract

Hepatocellular carcinoma (HCC) is the most common primary liver tumor, often arising in the context of chronic liver disease. Despite recent advances in systemic therapies, including the use of immune checkpoint inhibitors (ICIs), clinical outcomes remain suboptimal, with many patients exhibiting primary or acquired resistance. Accumulating evidence indicates that the dysregulation of epigenetic mechanisms contributes to HCC development, and may also play a crucial role in shaping the tumor immune microenvironment, influencing responses to treatments. In this study, we analyzed the expression profiles of a comprehensive set of epigenetic regulators across publicly available transcriptomic datasets of HCC and non-tumoral liver tissues. Our findings reveal a consistent dysregulation of key epigenetic modifiers, particularly those involved in DNA methylation and histone modification. Furthermore, our analysis underscores the need for a deeper understanding of the epigenetic landscape of HCC, as specific epigenetic patterns are directly associated with disease development, the major mutational, immune, and transcriptional subclasses of HCC, and patient clinical outcomes. Our study provides a foundation for integrating epigenetic biomarkers into patient stratification and therapeutic decision-making. A more comprehensive analysis of epigenetic alterations could pave the way for novel predictive markers and combination strategies that could enhance the efficacy of ICIs in HCC.

**Supplementary Information:**

The online version contains supplementary material available at 10.1007/s13105-025-01095-6.

## Introduction

Hepatocellular carcinoma (HCC) is the most common primary liver cancer and it is among the top three causes of cancer-related deaths in more than forty countries worldwide [18, 119]. Although the current incidence of HCC has declined globally since 2000 [128], the number of new cases per year is projected to increase significantly by 2040, when it is predicted that 1.3 million people could die from liver cancer [18, 118]. HCC normally develops on a background of chronic liver injury and inflammation caused by hepatitis B virus (HBV) or hepatitis C virus (HCV) infection, alcohol-induced liver disease and, in an increasing manner, by metabolic dysfunction-associated steatotic liver disease (MASLD)[91, 128]. Despite significant recent advances the prognosis of HCC patients remains poor, with a 5-year survival rate of only 18%, highlighting the limitations of current treatments [151]. When diagnosed early HCC can be treated by local ablation or resection; however, disease recurrence after three years occurs in 30–50% of patients due to intrahepatic metastases or de novo tumors emerging in the chronically injured liver parenchyma [12, 90]. For patients that are diagnosed at more advanced stages, systemic therapies are implemented. Targeted therapies with multikinase inhibitors such as sorafenib, lenvatinib, regorafenib, and cabozantinib pioneered the field and demonstrated positive, albeit modest, survival benefits [128]. The advent of immunotherapy with immune checkpoint inhibitors (ICIs)-antibodies that block the PD-1/PD-L1 pathway or CTLA-4 revolutionized systemic HCC therapy, and their use in combination with anti-VEGF antibodies has significantly improved the outcomes of patients with advanced HCC [89]. Moreover, ICIs are also actively being tested in the adjuvant and neoadjuvant settings in patients with early-stage disease in combination with surgery and local therapies [71, 90]. However, even with these advances, less than 30% of HCC patients achieve an objective response to ICI-based therapies [41]. Therefore, the identification of mechanisms of therapy resistance, biomarkers of response and new therapeutic targets is essential to improve HCC treatment and patients’ prognosis [149].

To advance HCC treatment, understanding the molecular and cellular bases of this tumor and those of the microenvironment on which it evolves is essential. The mutational landscape of HCC of different etiologies has been established over the past two decades [2, 22, 53, 115, 161]. Somatic mutations in the promoter of the telomerase reverse transcriptase gene (*TERT*) are the most frequent mutations found in HCC (30–60%), and can be observed in early steps of liver carcinogenesis [22]. HCCs of alcohol and HCV-related etiologies frequently harbor *TERT* promoter mutations, while in HBV-related HCC telomerase is usually upregulated through viral insertion in the *TERT* promoter [22]. Approximately 37% of HCC cases display activating mutations of *CTNNB1*, the gene coding for b-catenin, although these mutations are rarely found in dysplastic nodules [65]. On the other hand, the Wnt/b-catenin pathway can also be activated by mutations in the *AXIN1* or *APC* genes (15% and 2% of cases, respectively) [161]. Mutations of the tumor protein 53 (*TP53*) are detected in 20–50% of HCCs, with an incidence progressively increasing from early to advanced stages of the disease, and are more frequent in HBV-related cancers [22]. *TP53* and *CTNNB1* mutations tend to be mutually exclusive [121]. Less commonly, other cell-cycle related genes such as retinoblastoma (*RB1*) and cyclin dependent kinase inhibitor 2 A (*CDKN2A*) are mutated in tumors with poor prognosis [38]. Mutations in cell signaling-related genes are also less frequent, and include ribosomal protein S6 kinase (*RPS6KA3*) (2–10%), phosphatidylinositol-4,5-bisphosphate 3-kinase, catalytic subunit alpha (*PIK3CA*) (2%), *TSC1* and *TSC2* (3–8%), homozygous deletions of phosphatase and tensin homolog (*PTEN*) (2–3%), as well as amplifications of fibroblast growth factor 19 (*FGF19*) (5–10%) and vascular endothelial growth factor (*VEGF*) (4%) genes. Finally, mutations in genes related to the oxidative stress response such as nuclear factor erythroid 2-related factor 2 (*NFE2L2*), and kelch-like ECH-associated protein 1 (*KEAP1*) are observed in 6% and 4% of HCCs, frequently associated with mutations in *CTNNB1* or *AXIN1* [22]. These studies demonstrate the molecular complexity of HCC, and also highlight the fact that the most common mutations found in HCC are not directly actionable.

Starting twenty years ago several classification systems of HCCs based on transcriptomic data have been established. These classification systems have identified HCC subtypes associated with specific biological and molecular features of the tumors, such as proliferative activity, signaling pathways activation, differentiation status, mutational background and immune microenvironment status. Clinical characteristics, potential drug responses and patients’ outcomes are also captured by some of these classification schemes [21, 115]. The first study performed by Lee et al. established two transcriptomic subclasses that were related to differential patients’ survival [72]. This was followed by additional classification systems by Boyault et al. (G1-G6 subclasses) [16], Chiang et al. (five subclasses) [27], Hoshida et al. (S1-S3 subclasses) [63] and The Cancer Genome Atlas Research Network (TCGA) (iClust1-iClust3 subclasses) [2], and others [36, 117, 147]. Recent transcriptomic studies have also described different HCC subclasses according to the expression of immune-related genes. These subclasses reflect different landscapes of inflammatory cells infiltrates (i.e. immune inflamed, immune excluded and immune desert tumors) and identify patients with differential response to ICI-based therapy and survival [104, 127]. Altogether, these studies emphasize the value of comprehensive transcriptomic analyses for the understanding of HCC molecular build up.

Experimental evidence has cogently demonstrated the oncogenic potential of most of the genetic mutations described above, including observations in relevant murine models of HCC [103]. However, a role for epigenetic alterations in the remodeling of HCC transcriptome and tumor progression has recently become evident [10, 17, 46, 66], and epigenetic mechanisms are now recognized to contribute to most of the hallmarks of cancer cells [47, 58]. While intratumor heterogeneity is mostly studied under the light of genetic mutations, which can be present across all cancer cells or only in a subfraction of them, the existence of heterogeneous “cancer cell states” in cells with otherwise identical genetic background has gained attention [30]. Such cancer cell states are established by induced reversible transcriptional programs governing critical aspects of the neoplastic phenotype, which can be dictated to a great extent by epigenetic processes in cooperation with genetic drivers [58, 131]. Epigenetic gene regulation involves different mechanisms including DNA methylation, ATP-dependent nucleosome remodeling, the introduction of histone variants, post-translational modifications (PTMs) of histones, and non-coding RNAs (ncRNAs) [19, 112]. Contrary to genetic mutations, epigenetic mechanisms such as DNA and histones covalent modifications are highly flexible and dynamic, involving reversible enzymatic reactions and specific protein–protein interactions, which make them amenable to pharmacological intervention [8, 9, 44, 142]. Covalent epigenetic marks are introduced, removed and recognized by a broad set of proteins that according to these functions can be classified as epigenetic writers, erasers and readers [14, 46]. Relatively few mutations in chromatin remodeling genes (*ARID1A* and *ARID2*) as well as in histone modifying enzymes (*KMT2D*, *KMT2B* and *KMT2C*) have been described in HCC [161]. Besides mutations, changes in the expression of these epigenetic effector genes (EpiGs) are increasingly recognized to contribute to the neoplastic traits of tumor cells [30–32, 109], including HCC cells [7, 10, 138]. In the present study we have performed a comprehensive in silico transcriptomic analysis of the expression of EpiG in HCC tissues. Our data revealed EpiGs expression profiles directly associated with the development of the disease, with the major mutational, immune and transcriptional subclasses described above, and with the clinical evolution of the patients. These findings may improve our understanding of HCC pathogenesis and pave the way for the identification of novel adjuvant therapies.

## Materials and methods

### Functional selection of interrogated genes

Epigenetic genes were selected from the literature [14], *EpiFactors* [96], and *ChromoHub* [86] databases to generate a manually curated list. As we previously described [62], we included genes whose function was related to the methylation and acetylation of DNA and histones, the citrullination (or deimination) of histone arginine residues, and the readers of methyl and acetyl groups. The function of each selected gene was confirmed by the availability in reliable databases (GeneCards, PubMed and Uniprot) of experimental evidence demonstrating their purported biochemical activity. All the genes with no experimental evidence of functional activity were discarded. Reader and eraser functions were prioritized above reader or cofactor activity to classify the genes, although some of them have several functions as readers and erasers or writers [14].

### Data acquisition and preprocessing

Publicly available clinical information and gene expression profiles of patients was retrieved from their respective repositories. In the case of transcriptomic high-throughput (RNAseq) analyses data for GSE148355 [152], GSE114564 [129], GSE77509 [150], GSE111845 [20], GSE202069 [77] and GSE97098 [113] was downloaded from the NCBI Sequence read archive (SRA) in fastq format using SRA Toolkit Version 3.1.1. Adapter sequences and low-quality reads were removed using TrimGalore version 0.6.0 with Cutadapt version 1.18 [97]. Reads were subsequently aligned to the hg38 reference genome using the splice-aware aligner STAR (version 2.7.9a) [40]. Gene-level quantification was performed with STAR's quantMode GeneCounts option to count the number of reads mapped to each gene. To ensure consistent and reliable gene expression data, MANE annotations (version 1.4) [105] were utilized.

TCGA-LIHC gene expression data generated by the TCGA research network (https://www.cancer.gov/tcga) was retrieved as STAR-counts aligned to the hg38 genome, using TCGAbiolinks R package (version 2.34)[106] in R software Version 4.4.2 (hereafter called R). Clinical data for TCGA-LIHC were retrieved from the TCGA-CDR as outlined in the TCGA Pan-Cancer Clinical Data Resource (TCGA-CDR)[85]. Mutational data from the MC3 cohort was retrieved using TCGAmutations (version 0.4.0) [43]. Mutational data has been managed using maftools (version 2.22.0) [98] and represented using the oncoprint function from the ComplexHeatmap (version 2.22.0) package [54].

In the case of the ICGC LIRI-JP (International Cancer Genome Consortium Liver Cancer Japanese Project), controlled-acces gene expression, somatic mutation and clinical data corresponding to the 28 release was retrieved from the ICGC repository (http://platform.icgc-argo.org). For subsequent analysis, 40 liver cancer specimens were excluded, including 3 with metastatic tumors, 30 with ICC or cHCC/CC, and 7 with low tumor cell percentage or duplicated samples. For all RNA-seq datasets, including LIRI-JP, raw counts were normalized using the trimmed mean of M-values (TMM) using edgeR (version 4.4.1) [146]. TMM normalization accounts for library size differences and composition biases, ensuring accurate comparisons between samples.

On the other hand, microarray expression data for GSE61276 [15], GSE6764 [143], GSE14520-GPL3921 [117], GSE102079 [29], GSE89377 [125] were directly downloaded from the processed matrices available in the NCBI repository. Gene symbol annotations for each platform were updated using AnnotationDbi version 1.68.0 and org.Hs.eg.db version 3.20.0.

In the case of single-cell RNA sequencing (scRNAseq) data for primary fetal and adult human liver hepatocytes were downloaded as an AnnData object from the Cell Atlas: https://collections.cellatlas.io/liver-development, which corresponds to the processed samples of E-MTAB-8210 [139]. To facilitate downstream analyses, data were imported into the Scanpy framework (version 1.10.4) [140] using Python (Version 3.13.2). Following the import, the expression matrix and feature data were exported and subsequently processed using Seurat (version 5.2.1)[59]. The minimum number of cells required for inclusion was set to 100, and the minimum number of features per cell was set to 200 to ensure high-quality data. To ensure the integrity of the dataset, cells were filtered based on several quality control metrics. Specifically, cells with fewer than 500 detected features (genes), more than 20% mitochondrial gene expression, or predicted to be doublets were excluded.

### Pseudo-bulk RNAseq analysis

To perform pseudo-bulk RNA-seq analysis, samples with fewer than 50 cells were filtered out. Additionally, one adult sample was excluded as it originated from a separate study. Counts were aggregated by sample using the Seurat2PB function from edgeR, followed by a standard pseudo-bulk RNA-seq differential expression analysis pipeline. This pipeline involved typical RNA-seq preprocessing steps, including filtering and normalization of library sizes using TMM. For differential expression testing, the quasi-likelihood F-test was employed for each contrast. After filtering, three groups were formed based on previously defined developmental stages: one group labeled HB (hepatoblasts), comprising 3 samples from HB1 and HB2 stages (5–6 post-conception weeks, PCW), 7 samples from FH1 (fetal hepatocytes, 7–9 PCW), 4 samples from FH2 (12–17 PCW), and 3 samples from AH (adult hepatocytes).

### Molecular classification and expression analysis

To evaluate transcriptomic differences limma (version 3.62.2) [116] was used for microarrays. For RNAseq analysis, first, low expression genes were filtered using the *filterByExpr* function implemented in edgeR, with the default setting. After that the voom-limma pipeline for differential expression was used [116]. Previously described HCC molecular subclasses were identified for TCGA-LIHC and LIRI-JP datasets using the MS.liverK package (Petitprez, F. et al. 2013). For the assessment of the enrichment of specific genesets, single sample gene set enrichment analysis algorithm (ssGSEA) from the corto package (version 1.2.4) [101] was used. To visualize the expression data, heatmaps were generated using the package ComplexHeatmap.

### Sample clustering

Unsupervised clustering was performed separately on the tumoral samples from TCGA-LIHC and LIRI-JP datasets using Discriminant Analysis of Principal Components (DAPC) from the adegenet package (version 2.1.11) [68], using the *find.clusters* function. For each dataset, the optimal number of clusters was determined by first selecting the principal components (PCs) required to explain at least 95% of the variance. All linear discriminants were included, as the remaining eigenvalues were small enough to avoid significant differences in the analysis. The optimal number of clusters was chosen based on the"elbow"in the Bayesian Information Criterion (BIC) curve, where adding more clusters no longer significantly improved the model.

### Statistical analyses

Genes were considered differentially expressed if the adjusted p-value, calculated using the Benjamini–Hochberg FDR method, was below 0.05. For correlation analyses of gene expression data, Pearson’s correlation was used. Kaplan–Meier survival analyses were conducted using the survival package (version 3.8–3) in combination with the survminer package (version 0.5.0) for pairwise group comparisons. Multiple comparisons were controlled by the False Discovery Rate (FDR) using the Benjamini and Hochberg correction. Comparisons of continuous variables between two groups were performed using either Student's t-test or the Wilcoxon rank-sum test, depending on the distribution of the data. For pairwise comparisons, statistical significance was assessed using the Wilcoxon rank-sum test with Benjamini–Hochberg (BH) correction for multiple testing. A *p*-value < 0.05 was considered statistically significant.

## Results

### Landscape of epigenetic genes (EpiGs) expression in human HCC

**Fig. 1:**
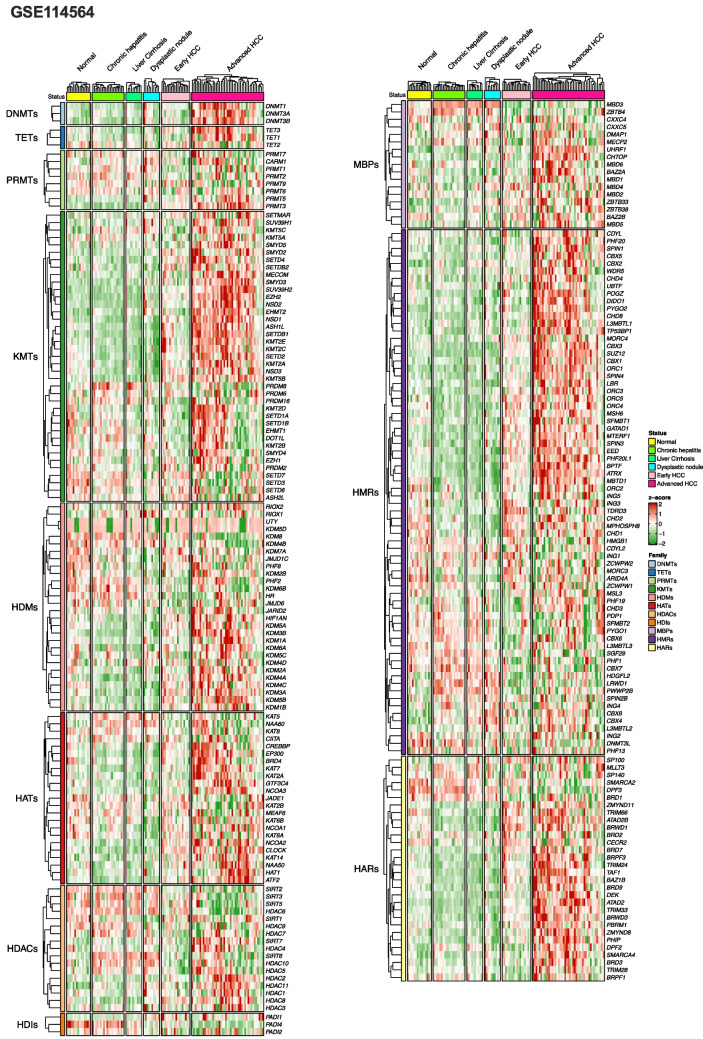
Heatmap of the expression of epigenetic genes grouped in families and according to liver disease classification (normal liver, chronic hepatitis, cirrhosis, dysplastic nodules and early and advanced HCC) in the GSE114564 dataset

**Fig. 2:**
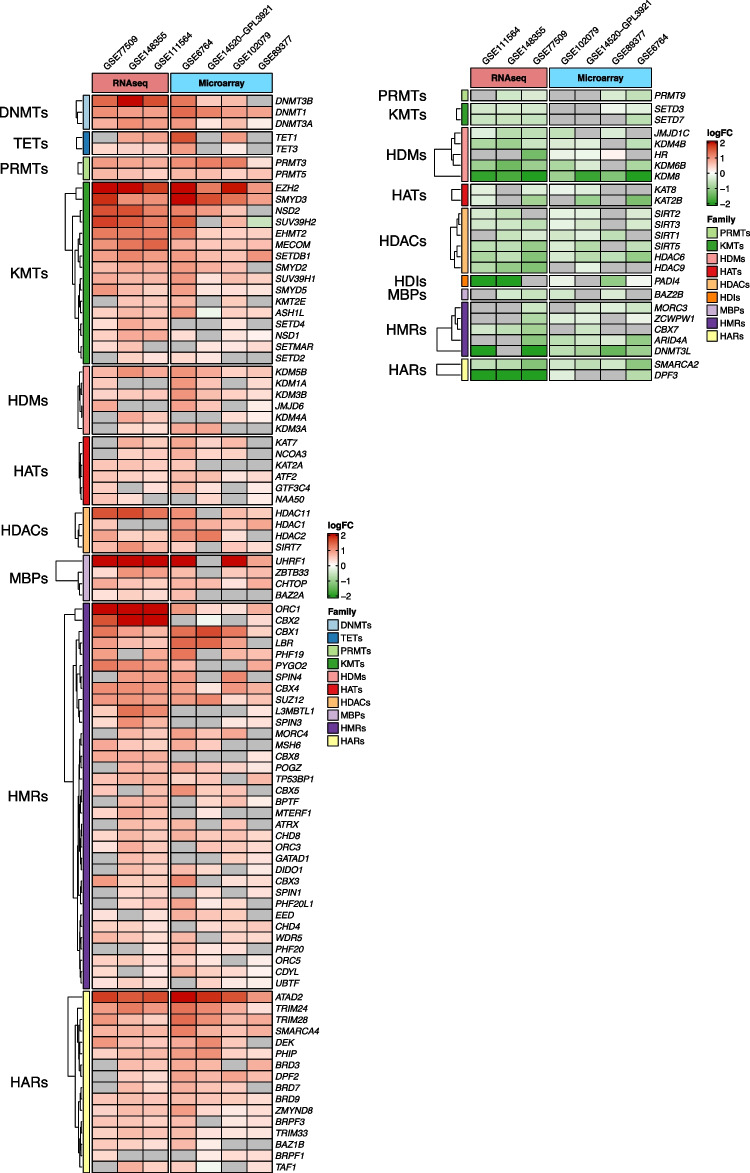
Epigenetic genes with statistically significant changes in expression between normal tissue and HCC from the indicated RNAseq and microarray datasets. Left panel, upregulated epigenetic genes; right panel, downregulated epigenetic genes

**Fig. 3:**
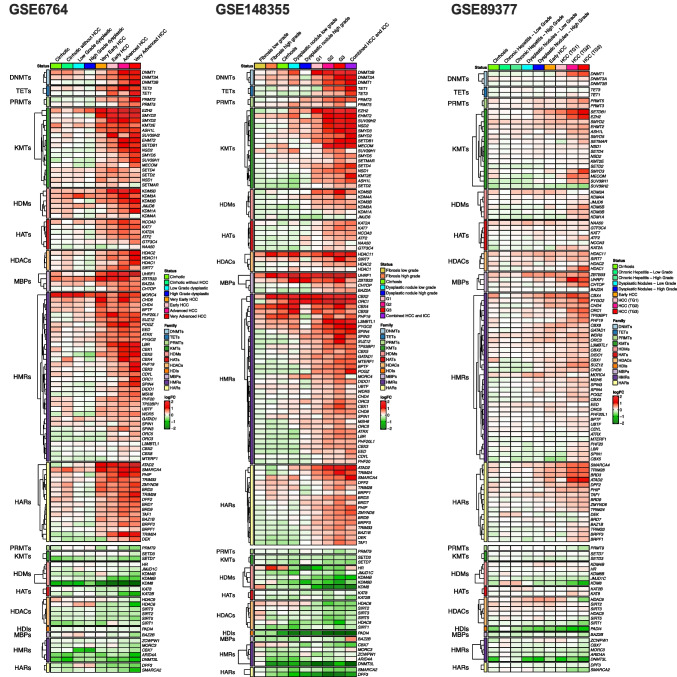
Changes in the expression levels of epigenetic genes at different stages of hepatocarcinogenesis in the different datasets indicated

We curated a list of 257 genes coding for epigenetic factors classified in 11 families, belonging to the three functional groups of epigenetic writers, erasers and readers (Table 1 and Suppl. Table 1), which we have previously verified that are expressed in the human liver [62]. The epigenetic writers included DNA methyltransferases (DNMTs), protein lysine-methyltransferases (KMTs), protein arginine-methyltransferases (PRMTs) and histone acetyl-transferases (HATs); epigenetic erasers were DNA demethylases (TETs), histone deacetylases (HDACs) histone-lysine demethylases (HDMs) and histone deiminases (HDIs); while epigenetic readers encompassed DNA methyl-binding proteins (MBPs), histone acetyl readers (HARs) and histone methyl readers (HMRs). In a first approach to explore the expression landscape of EpiGs in human HCC, we selected seven publicly available transcriptomic datasets with an ample representation of non-tumoral and tumoral tissue samples from patients with HCCs of different etiologies. Six of them also included tissue samples representative of different stages of the disease (Table 2). As can be appreciated in Fig. 1 and Suppl. Figure 1 A-C, significant changes in the expression of EpiGs were readily observed between normal and tumoral tissues in all datasets and across the different types of epigenetic writers, erasers and readers. In most cases there was an upregulation in EpiGs expression, although a subset of these genes was downregulated in tumoral tissues. The genes with statistically significant changes in expression between normal tissues and HCCs (FDR < 0.05) for each dataset are indicated in Suppl. Figure 2 A, B. When we compared non-tumoral tissues (total of 308 samples) with samples from the most advanced HCC stages (total of 280 samples) we identified 93 genes that were upregulated and 25 genes that were downregulated in at least four of the seven datasets analyzed (Fig. 2). The expression of some of these genes within this 118 gene signature, such as *DNMT1*, *UHRF1*, *EHMT2*, *EZH2*, *ATAD2*, *SMYD3*, *CBX4*, *SMARCA4*, *SMARCA2* and *KDM8* has been previously reported to be altered in HCC [7, 42, 46, 55, 81, 114, 137], which confirms the accuracy of our findings. Interestingly, changes in the expression levels of a number of genes within this set of 93 + 25 genes were already detected at pre-neoplastic or early stages of hepatocarcinogenesis (Fig. 3). This observation is in agreement with the notion that epigenetic modifications may precede the molecular alterations that drive human hepatocarcinogenesis [33, 37, 50], and suggest the potential early involvement of these genes in the tumorigenic process.

**Table 1:** Classification of Epigenetic genes

Class	Family	Full name	Genes
Writers	DNMTs	DNA methyltransferases	3
	PRMTs	Arginine (R) methyltransferases	9
	KMTs	Lysisne (K) methyltransferases	42
	HATs	Histone acetyl transferases	23
Erasers	TETs	Tet methylcytosine dioxygenases	3
	HDMs	Histone demethylases	29
	HDACs	Histone deacetylases	17
	HDIs	Histone deiminases	3
Readers	MBPs	Methylated CpG binding proteins	18
	HMRs	Histone methyl readers	77
	HARs	Histone acetyl readers	33

**Table 2:** Studies included for the analysis of human liver transcriptomic data from publicly available datasets

GEO ID	Type	Samples					
		**Fetal**	**Pediatric**			**Adult**	
GSE111845	RNAseq	10	10			10	
GSE61276	Microarray	10	-			10	
		**F1 (5–6 PCW)**	**F2 (7–9 PCW)**		**F3 (12–17 PCW)**	**Adult**	
E-MTAB-8210	scRNAseq	3	7		4	3	
		**Non-Tumor**	**Fibrosis**	**Chronic Hepatitis**	**Cirrhosis**	**Dysplastic Nodules**	**HCC**
GSE148355	RNAseq	16	20	-	10	17	65
GSE114564	RNAseq	15	-	20	10	10	63
GSE77509	RNAseq	20	-	-	-	-	40 (20)
GSE6764	Microarray	10	-	-	13	17	35
GSE102079	Microarray	14/91	-	-	-	-	152
GSE14520 (GPL3921)	Microarray	220	-	-	-	-	225
GSE89377	Microarray	13	-	20	12	22	40
GSE202069	RNAseq	-	-	-	-	-	17
TCGA-LIHC	RNAseq	50	-	-	-	-	371
LIRI-JP	RNAseq	197	-	-	-	-	203
		**Cell Lines**					
GSE97098	RNAseq	28					

PCW, post-conception weeks

### Oncofetal reprogramming of EpiGs expression in human HCC

**Fig. 4:**
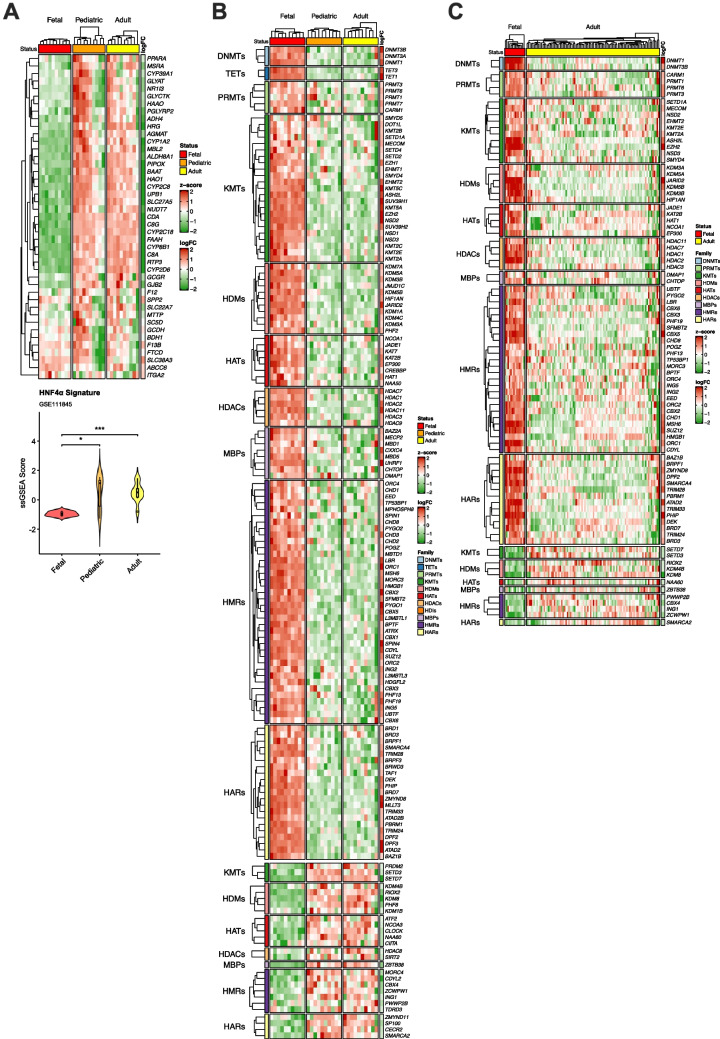
**A.** Heatmap of the expression of the 44-gene signature of HNF4α transcriptional activity in human liver tissue samples from fetal, pediatric and adult donors (GSE111845). Lower panel shows violin plots representing the ssGSEA of the HNF4α signature scores in the three different groups. *p* < 0.05 (*), *p* < 0.001 (***). **B.** Heatmap of the expression of epigenetic genes differentially expressed in fetal, pediatric and adult liver tissues (GSE111845) statistically significant. **C.** Heatmap of the expression of epigenetic genes differentially expressed in fetal, pediatric and adult liver tissues (GSE61276) statistically significant

As previously mentioned, one of the hallmarks of cancer is the phenotypic plasticity of transformed cells [30, 58]. Transcriptional reprogramming of tumor cells into stem-like cells with embryonic features, also known as onco-fetal reprogramming, is increasingly recognized to occur in cancer [95, 123], including HCC [66, 87]. Epigenetic mechanisms likely play a central role in the reactivation of genes characteristic of the fetal liver as well as in the repression of genes actively transcribed in the quiescent, differentiated and metabolically competent healthy adult liver [12, 66]. Hepatocyte nuclear factor 4 alpha (HNF4α) is a master regulator of hepatocyte differentiation and liver function [13, 110]. A recent study identified a 44-gene signature as an accurate read-out of HNF4α transcriptional activity in liver tissues, with high capacity to discriminate between advancing stages of both chronic liver disease and HCC [56]. We tested the expression of this signature in a transcriptomic RNAseq dataset (GSE111845) including human liver tissue samples from fetal, pediatric and adult donors [20] (Table 2). As could be expected we observed a consistent upregulation of most genes within this HNF4α signature in post-natal liver tissues (Fig. 4A). This finding was aligned with the expression of a complement of “liver-selective” genes characteristic of the adult healthy human liver, many of them involved in the synthesis of serum proteins and metabolic enzymes [64] (Suppl. Figure 3). Next, we examined the expression of our full complement of EpiGs and observed remarkable changes between fetal and pediatric or adult liver tissues. Most of these changes involved the downregulation of EpiGs belonging to all eleven categories, and the upregulation of a few specific genes upon liver maturation (Suppl. Figure 4 A). We identified a group of EpiGs with consistent expression changes between fetal and adult liver (adjusted *p*-value < 0.05). This signature included 125 and 27 genes that were downregulated or upregulated, respectively, in the adult human liver compared to the fetal liver (Fig. 4B). These global changes in EpiGs expression were also observed in a second dataset, a microarray analysis (GSE61276) of fetal and adult human liver tissues [15]. Of these 125 and 27 genes, 130 were detected in the GSE61276 microarray and 88 of them had a statistically significant differential expression between fetal and adult tissues (12 downregulated and 74 upregulated consistently with the findings in the GSE111845 dataset) (Fig. 4C). Importantly, most of these changes in EpiGs gene expression between fetal and adult liver that we observed in these two bulk-RNAseq studies were confirmed in a single-cell RNAseq transcriptomic analysis of human fetal and adult hepatocytes [139] (Suppl. Figure 4B). This suggests that the expression signal captured in the bulk RNAseq analyses would originate to a great extent from fetal and adult hepatocytes rather than from non-parenchymal cells.

**Fig. 5:**
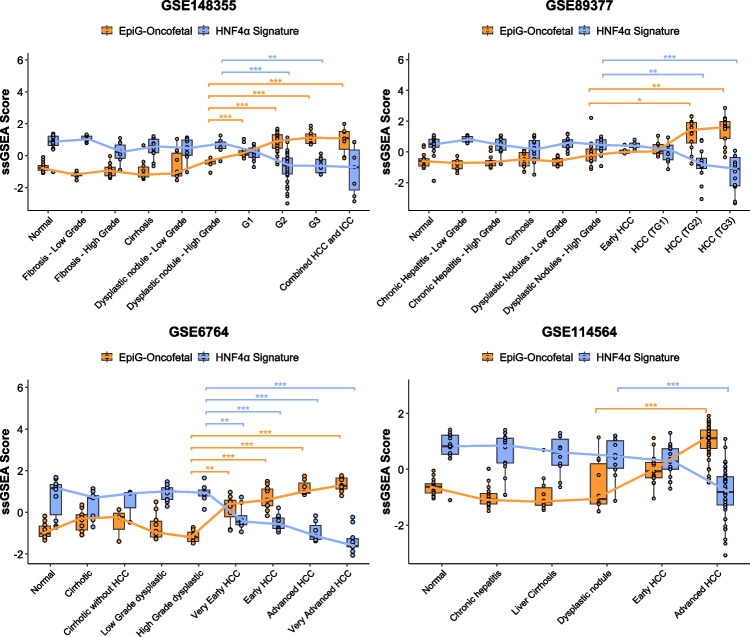
Boxplots of the EpiG-Oncofetal and HNF4α signatures analyzed using ssGSEA in normal liver tissues and tissues at different stages of hepatocarcinogenesis in the indicated genesets. Pairwise comparisons were performed using the Wilcoxon rank-sum test with Benjamini–Hochberg (BH) adjustment for multiple testing. The preceding stage before hepatocellular carcinoma (HCC) development was used as the reference group in all comparisons. Statistical significance is indicated as follows: *p* < 0.05 (*), p < 0.01 (**), p < 0.001 (***). Comparisons with p > 0.05 are not shown

Among the 125 genes that are highly expressed in the fetal liver 65 were found within the 93 genes that we previously identified as being upregulated in advanced HCC. On the other hand, of the 27 genes downregulated in the fetal *vs* adult liver there were 6 genes also downregulated in HCC samples compared to non-tumoral tissues (Suppl. Table 2). Next, we interrogated the enrichment of this “oncofetal” signature of 65 upregulated genes using ssGSEA in four datasets including samples corresponding to different stages of the disease, from normal liver to advanced HCC (GSE148355, GSE89377, GSE6764 and GSE114564, Table 2). We observed a progressive enrichment in this signature along the pathological stages of chronic liver disease and carcinogenesis, accompanied by a concomitant decrease in the expression of the HNF4α signature (Fig. 5).

### EpiGs gene signature identifies different HCC subclasses with distinct clinical outcomes and molecular phenotypes

**Fig. 6:**
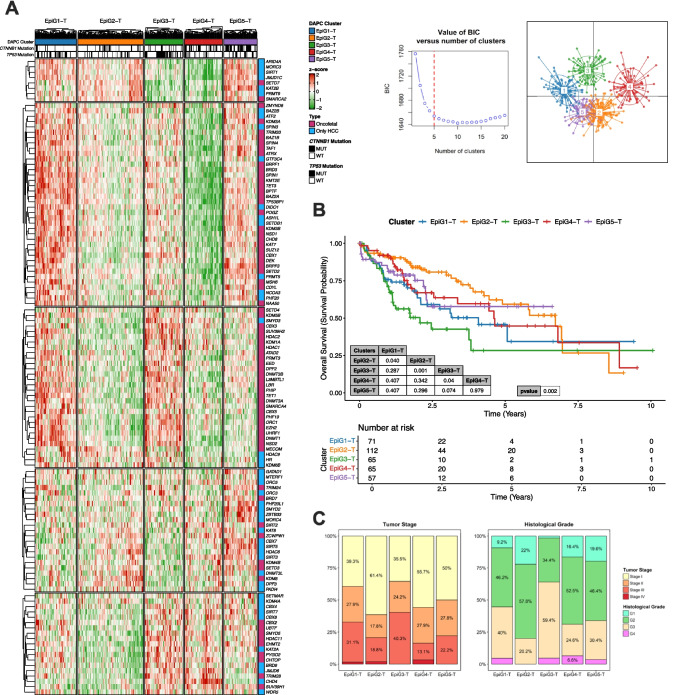
**A.** Heatmap identifying five subgroups (EpiG1-T – EpiG5-T) of patients based on an unsupervised hierarchical clustering analysis of the TCGA-LIHC cohort according to the expression of the EpiG signature (93 + 25 epigenetic genes), left panel. Elbow plot for optimal selection of the number of clusters, and scatterplot of unsupervised DAPC representing the centroid and ellipses of 95% confidence interval, right panels. **B.** Kaplan–Meier curves showing the differences in overall survival between the different EpiGT subgroups identified in the TCGA-LIHC cohort. **C.** Distribution of tumor samples among different tumor stages, stages I—IV (left plot), or histological grades, G1-G4 (right plot), within the different EpiGT subgroups

**Fig. 7:**
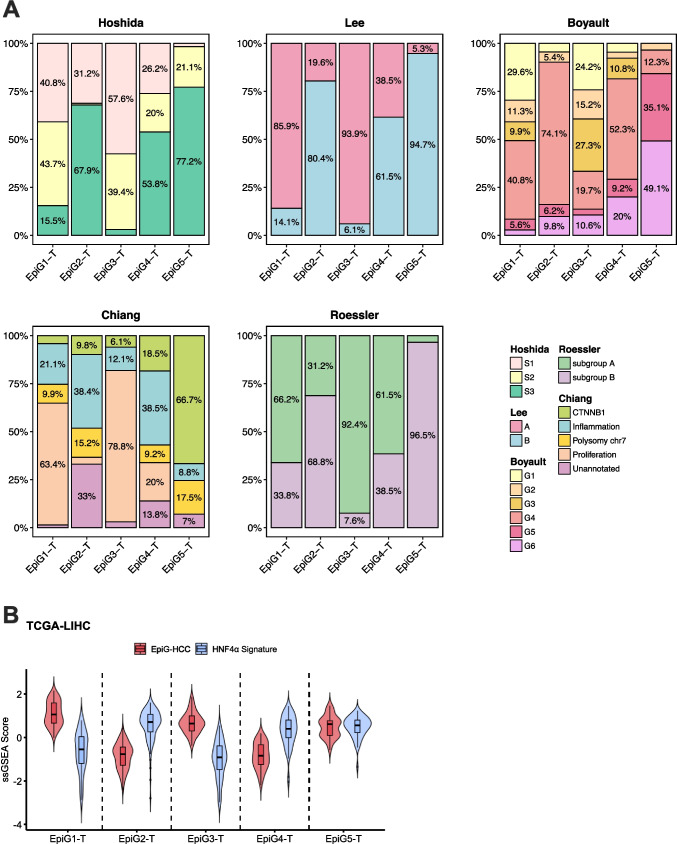
**A.** Distribution of tumor samples within each EpiGT subgroup according to the HCC subclassifications of Lee (A, B), Boyault (G1-G6), Chiang, Hoshida (S1, S2) and Roessler (A, B). **B.** Violin plots of the EpiG-HCC and HNF4α signatures analyzed using ssGSEA in the different EpiGT subgroups

While our analyses showed the widespread deregulation of EpiGs expression in HCC, they also hinted at the existence of different subgroups of patients with heterogeneous EpiGs expression profiles. To investigate if the differential expression of EpiGs could be related to the clinical and molecular characteristics of the tumors we performed an unsupervised hierarchical clustering analysis of patients in the TCGA-LIHC cohort [2] according to the expression of the 118 EpiG signature described above (93 upregulated and 25 downregulated genes in advanced HCC) (Fig. 6A). This signature clearly identified five subgroups (EpiG1-T to EpiG5-T) of patients, and these subgroups exhibited differential survival (Fig. 6B). Consistently, tumor stage and pathological grade were more advanced in the EpiG3-T subgroup, the one that showed the worst survival, compared to the other four EpiG-T subgroups (Fig. 6C). A detailed analysis of disease recurrence events in the TCGA-LIHC cohort revealed significant differences in disease-free interval (DFI) among the identified subgroups, indicating substantial variation in the timing of recurrence. Notably, EpiG3-T and EpiG1-T exhibited shorter median DFIs of 0.8 and 1.8 years, respectively, suggesting earlier recurrence (less than 2 years). In contrast, EpiG5-T, EpiG2-T, and EpiG4-T showed longer median DFIs of 2.1, 3.1, and 3.9 years, respectively, indicating later recurrence patterns (more than 2 years) [82] (Suppl. Figure 5A). At the molecular level, among all subclasses, tumors in the EpiG3-T group showed the highest frequency of mutations in the *TP53* gene (66%) (Suppl. Figure 5B). Moreover, this EpiG3-T group was enriched in tumor samples classified in the S1 and S2 Hoshida subclasses [63], the Lee subclass A [72], Boyault G1-G3 subclasses [16], the subgroup A of Roessler [117], and the proliferation class of Chiang [27], which encompass transcriptional and mutational profiles associated with worse clinical prognosis (Fig. 7A). A similar molecular profile was observed for the EpiG1-T subgroup and, consistently, patients included in this cluster also showed a poor overall survival (Fig. 7A and 6B). Interestingly, analysis of the HNF4α target gene signature revealed that patients in the EpiG3-T and EpiG1-T subgroups of the TCGA-LIHC cohort displayed the lowest levels of HNF4α activity (Fig. 7B). We performed an equivalent analysis in a second well-characterized cohort of HCC patients, the LIRI-JP cohort http://platform.icgc-argo.org [48]. Unsupervised hierarchical clustering analysis of patients’ HCC samples in the LIRI-JP cohort, according to the expression of the 118 (93 + 25) EpiG signature, also identified five subgroups of patients with differential survival (EpiG1-L to EpiG5-L) (Suppl. Figure 6). Patients in the EpiG4-L group had the worst outcome, and the pathological and molecular features of their tumors according to their mutational and transcriptional profiles, including the HNF4α target gene signature, were similar to those in the EpiG3-T group (Suppl. Figure 7 A-E). The clinical characteristics of patients allocated to the EpiG subgroups established in the TCGA-LIHC and LIRI-JP cohorts are described in Suppl. Table 3.

In order to better understand the role of EpiGs in HCC pathogenesis we searched for those genes that were simultaneously upregulated or downregulated in the EpiG clusters with worse clinical and molecular characteristics, the EpiG3-T and EpiG4-L subgroups. We identified 68 and 22 genes that were up- or downregulated, respectively, in both subgroups compared to non-transformed liver tissues. We found genes belonging to all EpiG functional categories (Suppl. Table 4). A preliminary indication of the relevance of these upregulated EpiGs as therapeutic targets in HCC was obtained by accessing data of a CRISPR/Cas9 dropout screen in human HCC cell lines (n = 21) (https://score.depmap.sanger.ac.uk/) [11]. We observed that the growth of HCC cell lines was significantly impaired upon genetic inactivation of 33 of these 68 genes (Suppl. Figure 8A). In addition, we analyzed transcriptomic data available from 28 HCC cell lines representing the molecular diversity of HCC [113]. A supervised classification was performed based on 3 robust transcriptomic subgroups of liver cancer cell lines driven by the differentiation state and sharing features similar to those described in HCC tumors, previously reported [23]. The first CL1 subgroup, described as “hepatoblast-like,” express hepato-specific genes and fetal/progenitor markers, corresponding to the defined “progenitor subclass” of HCC [88]. In contrast, the CL2 (“mixed epithelial–mesenchymal”) and CL3 (“mesenchymal-like”) subgroups are less differentiated and show activation of the TGF-β and noncanonical β-catenin signaling pathways. These two subgroups resemble the “Wnt–TGF-β” subclass [63] and Boyault’s G3 subclass [16]. Notably, we observed distinct differential expression patterns of epigenetic effectors across the three subgroups CL1, CL2 and CL3, consistently distributed again across all EpiG functional categories (Suppl Fig. 8B).

Next, to gain a more detailed insight into the EpiG transcriptomic landscape of the different subgroups we conducted pairwise comparisons among them. We identified those EpiGs that were specifically upregulated or downregulated in each group (Table 3). Interestingly, among the upregulated EpiGs we observed a significant overlap between EpiG5-T and EpiG1-L, subgroups with higher frequency of mutations in the *CTNNB1* gene (see below), and also between EpiG1-T and EpiG4-L, both enriched in samples with molecular characteristics related to a bad prognosis (Lee Subclass A and Hoshida subclasses S1 and S2) and including patients with very poor overall survival.

**Table 3:** List of genes specifically upregulated or downregulated in each EpiG subgroup

TCGA-LIHC		EpiG1-T	EpiG2-T	EpiG3-T	EpiG4-T	EpiG5-T
	Up	*ASH1L, BAZ2A, BAZ2B, BPTF, BRD3, CBX3, CHD8, DEK, DIDO1, DNMT1, GTF3C4, KAT7, KDM3B, KMT2E, MSH6, NAA50, NCOA3, NSD1, NSD2, PHIP, POGZ, SETDB1, SPIN1, SPIN3, SPIN4, SUZ12, TET1, TET3, TP53BP1, TRIM33* **[*n* = 30]**	*DPF3, KDM8* **[*n* = 2]**	*BRD9, CBX3, EED, HDAC1, HDAC2, KAT2A, KDM1A, L3MBTL1, PHF19, PYGO2, SETD4, SMYD3* **[*n* = 12]**	*CHD4, JMJD6, SIRT2, ZCWPW1* **[*n* = 4]**	*BRD7, BRPF3, CBX7, GATAD1, HDAC6, KAT2B, MORC4, PHF20L1, PRMT5, SETD3, SIRT1, SIRT5, SMYD2, TRIM24, ZBTB33* **[*n* = 15]**
	Down		*BRD9, CBX3, CBX4, CHTOP, DNMT3A, DPF2, EHMT2, EZH2, HDAC11, JMJD6, KAT2A, KDM1A, PYGO2, SMYD3, SMYD5, SUV39H1, SUV39H2, TRIM28, UBTF, WDR5* **[*n* = 20]**	*DPF3, HDAC6, KDM8, SETD7, SIRT5* **[*n* = 5]**	*ARID4A, ASH1L, ATF2, ATRX, BAZ1B, BAZ2A, BAZ2B, BPTF, BRD3, BRPF1, BRPF3, CBX3, CHD8, DEK, DIDO1, DNMT1, DNMT3B, GTF3C4, JMJD1C, KAT7, KDM3A, KDM3B, KDM6B, KMT2E, L3MBTL1, MORC3, MSH6, NAA50, NCOA3, NSD1, NSD2, ORC3, PHF20, PHIP, POGZ, PRMT3, PRMT5, SETD2, SETDB1, SIRT1, SPIN1, SPIN3, SPIN4, SUZ12, TAF1, TET3, TP53BP1, TRIM33* **[*n* = 48]**	
LIRI-JP		**EpiG1-L**	**EpiG2-L**	**EpiG3-L**	**EpiG4-L**	**EpiG5-L**
	Up	*ASH1L, BRPF3, CBX3, CBX7, KAT2B, KDM3B, MORC4, PHF20, PHF20L1, PRMT5, SETD7, SIRT1, SIRT5, SMYD2, TAF1, ZBTB33* **[*n* = 16]**	*JMJD6, SIRT2* **[*n* = 2]**	*BAZ2B, JMJD1C, MECOM* **[*n* = 3]**	*ATAD2, BRPF1, CBX1, CBX2, CBX3, CBX5, CHD4, CHD8, DIDO1, DNMT1, DNMT3A, DPF2, EZH2, HDAC2, NCOA3, ORC1, SMARCA4, SPIN1, SPIN4, SUV39H2, SUZ12, TET1, UHRF1* **[*n* = 22]**	*DNMT3L, HDAC6, KAT2 A, KDM4B, KDM8, ORC3, SETD4, SIRT7, ZMYND8* **[*n* = 9]**
	Down	*HDAC9, HR, TET1* **[*n* = 3]**	*ARID4A, ASH1L, BAZ2A, BAZ2B, BPTF, BRPF1, CHD8, DPF3, KDM3 A, KDM3B, KDM6B, KMT2E, MORC3, NSD1, PHIP, SETD2, SPIN1, SPIN3, SPIN4, TAF1, TET3, TP53BP1, TRIM33, ZMYND8* **[*n* = 24]**	*BRD9, CBX8, CHTOP, EHMT2, KAT2A, PYGO2, SIRT7, SMYD5, SUV39H1, TRIM28, UBTF, WDR5* **[*n* = 12]**	*SIRT2* **[*n* = 1]**	*BRD7, CBX1, CBX3, CDYL, DEK, DPF2, GTF3 C4, HDAC2, KAT7, MSH6, NAA50, ORC5, PHF20, PRMT5, SMYD3, SUV39H2* **[*n* = 16]**

### EpiG gene expression and HCC immune landscape

**Fig. 8:**
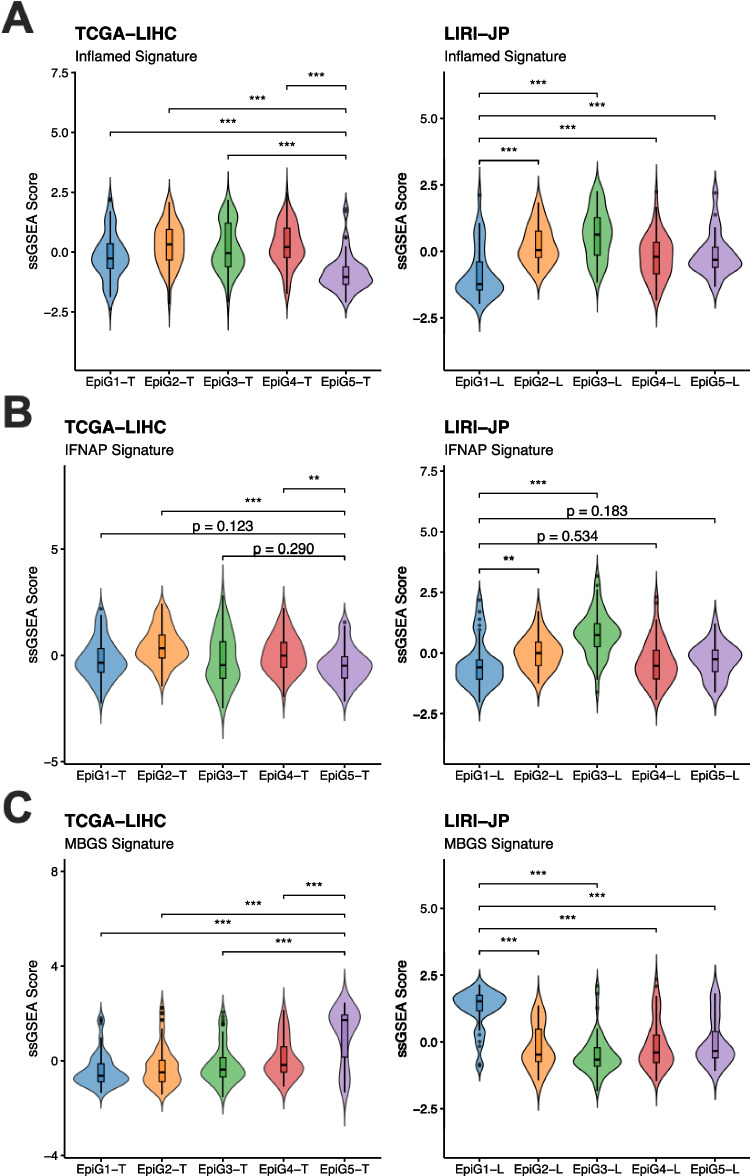
**A.** Violin plots showing the inflamed signature. **B.** Violin plots showing the IFNAP signature. **C.** Violin plots showing the Mutated b-catenin Gene Signature (MBGS), as analyzed using ssGSEA in the different EpiGT (TCGA-LIHC) and EpiGL (LIRI-JP) subgroups. *p* < 0.001 (***). For comparisons not reaching statistical significance (*p* > 0.05), exact *p*-values are reported

ICIs in combination with anti-VEGF antibodies are currently the first-line therapy for HCC systemic treatment [28, 89]. However, in advanced disease only about one-third of patients show an objective response [28]. Understanding the characteristics that define tumor immunogenicity, and thus predict the response to ICI-based therapies, is still an unmet need [120]. Immunogenomic classifications have established the existence of a robust 20-gene signature, called the “inflamed signature”, that identifies a so-called inflamed class of tumors likely to respond to ICI therapy [61, 76, 104]. In addition, an 11-gene signature, termed IFNAP, capable of predicting response and survival in patients with advanced HCC treated with anti-PD1 antibodies in frontline was also recently elucidated [57]. Epigenetic changes in tumoral and immune cells are increasingly being involved in the response of HCC tumors to ICI-based therapies [67, 93, 133]. Therefore, we evaluated the expression of both the “inflamed signature” and the IFNAP signature in the EpiG subgroups that we had identified in the TCGA-LIHC and LIRI-JP cohorts. In both cohorts we detected an EpiG subgroup, EpiG5-T and EpiG1-L in TCGA-LIHC and ICGC-LIRI-JP, respectively, that showed significantly reduced expression of the “inflamed signature” compared with all other subgroups (Fig. 8A). The expression of the IFNAP signature was also lower in these two subgroups when compared to most of the other subgroups (Fig. 8B). These observations indicate that a subset of HCC patients characterized by their EpiG expression profile would be less responsive to ICI therapies. Different experimental and human studies have demonstrated a strong association between the presence of *CTNNB1* mutations, the activation of the Wnt/b-catenin pathway, immune exclusion, and potentially a poor response to immunotherapy [45, 49, 60]. A recent study developed a transcriptomic signature able to predict *CTNNB1* mutational status and Wnt/b-catenin pathway activity, named the Mutated b-catenin Gene Signature (MBGS) [74]. We analyzed the expression of this MBGS signature across our EpiG subgroups observing significantly increased scores in EpiG5-T and EpiG1-L, precisely those with higher frequency of mutations in the *CTNNB1* gene (Fig. 8C). Importantly, these were the EpiG subgroups with lower expression of the “inflamed signature” and the IFNAP signature (Fig. 8A and B). These observations are consistent with the frequent association between Wnt/b-catenin pathway activation and immune paucity and exclusion in HCC tumors [104].

### Identification of potential epigenetic targets in HCCs with activated Wnt/b-catenin pathway

**Fig. 9:**
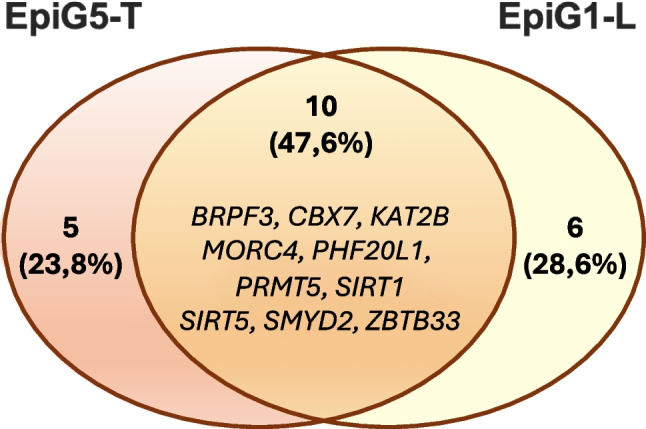
Venn diagram showing the number of EpiGs specifically upregulated in the EpiG5 T and EpiG1-L subgroups, and the number and identity of EpiGs commonly upregulated in both subgroups

The relevance of the Wnt/b-catenin signaling pathway in hepatic tumorigenesis makes it a potential therapeutic target. However, directly targeting b-catenin with small molecules is challenging, and despite many efforts no specific therapies have been approved so far [5, 65, 108]. Alternatively, disruption of oncogenic mechanisms that cooperate or are positioned downstream of the Wnt/b-catenin pathway may offer therapeutic opportunities [5, 65]. Epigenetic mechanisms involve reversible biochemical modifications and protein interactions amenable to pharmacological intervention, and a variety of “epidrugs” are actively being developed with promising preclinical antitumoral results [34]. With this in mind, we searched for those genes in our 118 (93 + 25) EpiG signature that were specifically upregulated in both the EpiG5-T and EpiG1-L subclasses compared to the other EpiG subgroups. There were 37 genes upregulated in EpiG5-T and 22 genes upregulated in EpiG1-L, and 13 were common between these two subgroups (Fig. 9). These genes belonged to different EpiG functional families, and many of them had been previously described to participate in Wnt/b-catenin biological activity in different tumor cell types [35, 69, 70, 78, 82, 94, 99, 111, 130, 135, 153, 154], suggesting their potential involvement in Wnt/b-catenin-driven hepatocarcinogenesis.

**Fig. 10:**
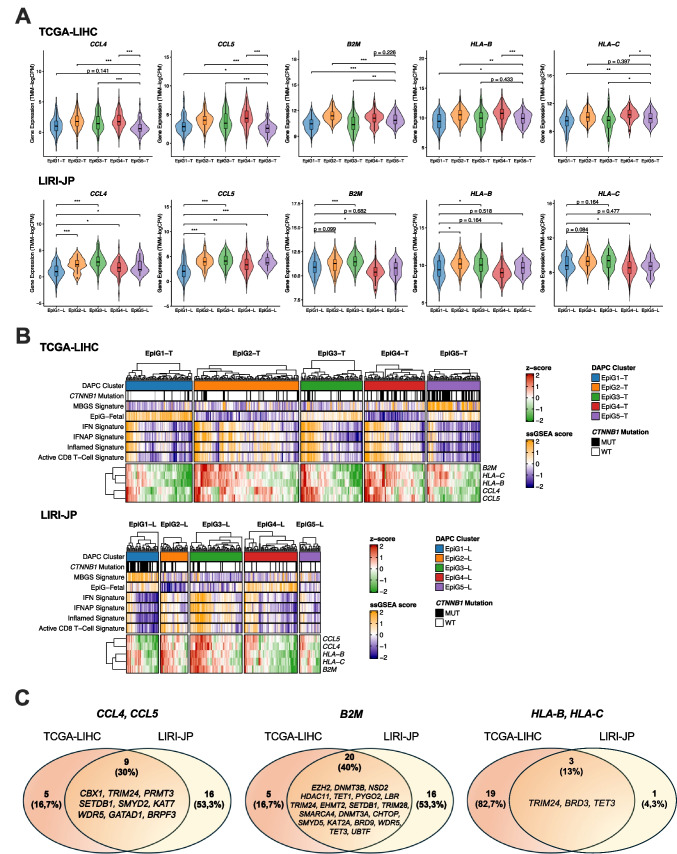
**A.** Violin plots showing the expression of five immune-related genes (*CCL4*, *CCL5*, *B2M*, *HLA-B* and *HLA-C*) across the five EpiG subgroups identified in the TCGA-LIHC, upper panel and LIRI-JP, lower panel, cohorts. *p* < 0.05 (*), *p* < 0.01 (**), *p* < 0.001 (***). For comparisons not reaching statistical significance (*p* > 0.05), exact p-values are reported. **B.** Heatmap showing the expression of the indicated immune response-related genes in the different EpiG subgroups of the TCGA-LIHC and LIRI-JP cohorts. *CTNNB1* mutational status and the expression of different immune-related signatures, as well as that of the fetal EpiGs (the 125 genes that are upregulated in the fetal vs adult liver shown in Fig. 4B) are also shown. **C.** Venn diagrams showing the number of EpiGs that show a statistically significant inverse correlation (R < −0.25) in their expression levels with that of the immune-related genes indicated in the TCGA-LIHC and LIRI-JP cohorts. The number and identity of the EpiGs that display this negative correlation simultaneously in both cohorts are also shown

As mentioned above, activation of the Wnt/b-catenin pathway is apparently associated with a poorer response to ICI-based therapies [49, 57, 60, 74]. Wnt/b-catenin driven immune exclusion in HCC is thought to involve the downregulation of chemokines driving dendritic cells and lymphocyte chemotaxis such as *CCL4* and *CCL5*, as well as the epigenetic repression of genes implicated in antigen type I presentation like *B2M*, *HLA-B* and *HLA-C* [104]. With this in mind, we evaluated the expression of these immune-related genes in the EpiG subclasses identified in the TCGA-LIHC and LIRI-JP cohorts. As shown in Fig. 10A, in agreement with the *CTNNB1* mutational status, the MBGS score, and a reduced activity of previously described IFN and activated CD8 T-cells signatures [4, 26] (Fig. 8C and 10B), the expression of these genes was predominantly lower in the EpiG5-T and EpiG1-L subclasses. Interestingly, the EpiG1-T and EpiG4-L subgroups, which do not display Wnt/b-catenin pathway activation, also tended to have lower levels of expression of immune-related genes (Fig. 10 A and B). To identify epigenetic genes that could be potentially involved in their repression we searched for EpiGs showing a statistically significant inverse correlation (R < −0.25) in their expression levels with that of the immune-related genes. We identified a number of EpiGs that showed such inverse correlation both in the TCGA-LIHC and LIRI-JP cohorts (Fig. 10C). Similarly, we evaluated the expression of EpiGs in seventeen patients from dataset GSE202069, all of whom had available clinical response data to anti-PD-1 therapy and biopsy or surgical resection specimens (8 responders and 9 non-responders). Despite the limited cohort size, we were able to identify 13 specific epigenetic effectors that were significantly upregulated in non-responder patients (Suppl. Figure 9). Hypothetically, the pharmacological targeting of these EpiGs could be leveraged to increase the efficacy of HCC immune therapy.

## Discussion

Epigenetic alterations are increasingly recognized to participate in HCC development already from the early pre-neoplastic stages during chronic liver injury. Our understanding of the consequences of specific changes in DNA methylation and histones post-translational modifications, including their impact on the expression or repression of key cancer-related genes, has significantly increased in recent years [17, 84]. However, the mechanisms responsible for the dysregulation of the epigenetic machinery are less well-known. Mutations in EpiGs are not frequently found in HCC [161], albeit the frequency of these alterations may be underestimated [10]. Therefore, it is likely that other processes would be involved. Fluctuations in the cellular levels of metabolites that are substrates of epigenetic writers and erasers can effectively modulate the epigenome and thus contribute to disease development and cancer [25, 51, 134]. The impact of these metabolic alterations on the epigenetic machinery may be particularly relevant in liver disease, given the profound rewiring of central metabolic pathways that occur in hepatocarcinogenesis, and deserves further consideration [6, 12, 62, 75, 92]. Another little-explored mechanism of epigenetic regulation likely to be altered in disease would be the cell signaling pathways that via phosphorylation and dephosphorylation reactions control the activity of epigenetic writers and erasers [122]. In the present study we addressed an additional mechanistic aspect of the epigenetic dysregulation in HCC by taking a holistic and comparative view to EpiG gene expression in hepatocarcinogenesis. Previous works reported the altered expression, and in some cases the potential role, of specific epigenetic genes in HCC [46, 84, 132]. Here we performed what we believe is a most comprehensive analysis of EpiGs expression in HCC tissues. We observed significant alterations, mostly upregulation, in the expression of genes belonging to all different categories of epigenetic genes. We identified a complement of 118 EpiGs which expression was consistently altered in transformed tissues. As previously mentioned, several of the identified epigenetic modifiers such as *DNMT1, UHRF1, EHMT2 or EZH2* were known to be altered in HCC. Other EpiGs highly induced in tumor tissues, such as *HDAC11* [24, 160] and *CBX2* [80, 136, 141, 158], are attracting significant attention as chromatin regulators capable of predicting prognosis and therapeutic responses. It is interesting to note that the expression of certain epigenetic modifiers is specifically altered in established tumors. Among these EpiGs were *BRPF3, TAF1, BAZ1B* or *DEK*, histone acetyl readers about which there is little information regarding their involvement in HCC pathobiology [73, 148]. However, in many other cases global changes in EpiGs expression were already observed in chronically injured liver tissues and early stages of HCC, such as the increasing induction of *EZH2, KDM5B, KDM3A, ZBTB33*, *MORC4* and *SMARCA4*, or the progressive downregulation of *KDM8, ARID4A, DNMT3L* and *SMARCA2*. These observations further support the notion that epigenetic alterations amplified in cancer can emerge before the onset of tumors [3, 12, 33, 50].

Most interestingly, we also identified EpiGs that were highly expressed during fetal liver development, were downregulated upon maturation and that became induced in tumoral tissues. We believe that this phenomenon may play a central role in the onco-fetal reprogramming of gene expression that occurs in cancer, when tumor cells acquire phenotypic characteristics of early developmental programs, including stem cell-like properties, that contribute to malignant transformation [87, 124]. Importantly, a number of these onco-fetal EpiGs are amenable to pharmacological targeting [34]. Therefore, their inhibition could be hypothetically harnessed to revert HCC cells dedifferentiation and suppress their malignant phenotype [159]. On the other hand, although less numerous, there was a subset of EpiGs that were upregulated in the transition from the fetal to the adult liver and subsequently were repressed in HCC tissues. Among these EpiGs were the lysine demethylases *KDM8* and *KDM6B*. These genes are known to be induced in different solid tumors in association with poor patients’ prognosis, while their repression in HCC is linked to poorer survival [1, 79]. We also found that the expression of several EpiGs, also including *KDM8* and *KDM6B*, gradually declined along the progression of chronic liver disease towards HCC. These observations suggest that certain epigenetic effectors could play an important role in the establishment of the mature and quiescent liver phenotype and in the preservation of liver function [12].

Our unsupervised hierarchical clustering analysis using the 118 genes EpiG signature identified five subgroups in the TCGA-LIHC cohort. Two of these subgroups, EpiG1-T and EpiG3-T, showed the worst overall patients’ survival, reduced time to recurrence and consistently were associated with the most aggressive molecular subclasses according to Boyault [16], Chiang [27], Hoshida [63] and Roessler [117] classifications. Interestingly, while the EpiG1-T and EpiG3-T subgroups had very similar aggressive molecular profiles and a poor prognosis they clustered differently according to their EpiGs expression profile. Currently we do not have an explanation for this divergence, although we noticed significant differences in the etiology of HCC patients between these two subgroups, with higher prevalence of alcohol-related liver disease in patients within EpiG3-T than in EpiG1-T. An equivalent unsupervised clustering analysis was performed in the LIRI-JP cohort, where we also identified five EpiG subgroups. In this case we also identified a subgroup, EpiG4-L, as that with a more aggressive molecular profile and poorest overall patients’ survival. We observed a significant overlap among the epigenetic genes specifically upregulated in EpiG4-L and the also very aggressive EpiG1-T subgroup from the TCGA-LIHC cohort. We have highlighted the EpiGs that were specifically upregulated in these subgroups with the intention of potentially guiding future mechanistic research to tackle aggressive HCCs. By integrating genomic, transcriptomic, and proteomic profiling across multiple panels of HCC cell lines, strong similarities with established HCC molecular subclasses have been established [23]. Moreover, the distinct and subgroup-specific epigenetic landscapes observed in these cell lines can provide novel insights into the complex interplay between epigenetic effector expression, cellular differentiation states, genetic alterations, and potentially drug responses.

One very interesting outcome of our EpiG clustering analyses was the identification of subgroups of HCCs with different immune landscapes, both in the TCGA-LIHC and the LIRI-JP cohorts. Expression of the “inflamed signature” and the IFNAP signature was lowest in EpiG5-T and the EpiG1-L, suggesting that patients in these subgroups would be less responsive to ICI-based therapies [57, 104]. In agreement with previous reports [74, 104] these subgroups were enriched in *CTNNB1* mutations and showed enhanced Wnt/b-catenin pathway activity. Intriguingly, in this analysis we also detected other two subgroups, EpiG1-T and EpiG4-L, in which the expression of immune response-related genes was also depressed like in the *CTNNB1* mutations enriched subgroups (EpiG5-T and EpiG1-L), but in the absence of Wnt/b-catenin pathway activation. Therefore, there must be additional mechanisms besides the activation of this signaling pathway that may drive immune exclusion. Although we do not have an explanation for this variation, we also noticed that the EpiG1-T and EpiG4-L subgroups were particularly enriched in the expression of EpiGs characteristic of the fetal liver. In this sense it is interesting to recall that fetal tissues are characterized by an immunosuppressive ecosystem, and that onco-fetal reprogramming is being increasingly associated with an immunosuppressive tumor microenvironment [83].

In view of the still limited proportion of patients that benefit from ICI therapies, one active area of research is the modulation of antitumor immunity with epigenetic drugs to enhance the efficacy of ICIs [84, 93, 133]. In our study we pinpointed several epigenetic genes that were selectively upregulated in the EpiG5-T and EpiG1-L non-inflamed subgroups. Noteworthy, for some of these epigenetic effectors, such as *NSD2* and *PRMT5*, there are small molecule inhibitors currently being tested in clinical trials for solid and hematological malignancies [34]. In the same vein, we also identified a set of EpiGs inversely correlating with the expression of genes involved in lymphocyte chemotaxis and in antigen presentation that are consistently downregulated in non-inflamed HCCs [104] or EpiGs significantly downregulated and upregulated in patients who do not respond to immunotherapy. It could be speculated that pharmacological interference with these EpiGs could also change the inflammatory landscape of these tumors and restore their immune sensitivity. There are inhibitors that have been tested in experimental models for many of them, such in the case of those inhibiting *SMYD2* [100, 107], *KAT2B* [102] or *SIRT5* [144]. For some of these EpiGs, such as *EZH2* [145] and *PRMT3* [39], the efficacy of therapeutic strategies involving their inhibition alongside ICIs administration has already been tested. Meanwhile, there are evidences suggesting that other EpiGs like *SMARCA4*, *SETDB1, SIRT5* or *CECR2* [126, 155–157] may be useful as predictive markers of response to immunotherapy and as pharmacological targets for combinatory approaches.

In summary, we have provided a holistic epigenetic portrait of HCC from a transcriptomic perspective. We believe that our study will help to better understand important aspects of liver carcinogenesis, and to identify new avenues to improve the therapeutic management of a tumor with limited treatment options.

## Supplementary Information

Below is the link to the electronic supplementary material.

ESM 1 Supplementary file1 (XLSX 40 KB)

ESM 2 A (PNG 1.56 MB)

High Resolution Image **Supplementary Figure 1.** **A**-**C.** Heatmaps of the expression of EpiGs classified in their different families in the indicated genesets. (EPS 19.4 MB)

ESM 2B (PNG 722 KB)

High Resolution Image (EPS 4.46 MB)

ESM 2 C (PNG 988 KB)

High Resolution Image (EPS 7.42 MB)

ESM 3 A (PNG 473 KB)

High Resolution Image **Supplementary Figure 2.** **A **and** B.** Dot plots identifying the EpiGs that show statistically significant changes in expression between normal liver tissues and HCC tissues in the indicated datasets. (EPS 246 KB)

ESM 3B (PNG 307 KB)

High Resolution Image (EPS 201 KB)

ESM 4 (PNG 486 KB)

High Resolution Image **Supplementary Figure 3.** Heatmap of the expression of liver-selective genes characteristic of the adult healthy liver (HSIAO signature) across fetal, pediatric and adult liver tissues (GSE111845). Right panel shows violin plots representing the ssGSEA analysis of the HSIAO signature scores in the three different groups. *p *< 0.001 (***). (EPS 1.18 MB)

ESM 5 (PNG 578 KB)

High Resolution Image **Supplementary Figure 4.** **A.** Heatmap of the expression of all EpiGs across fetal, pediatric and adult liver tissues (GSE111845). **B.  **Heatmap showing the scRNA-seq analysis of EpiGs expression in hepatoblasts (HB) (5-6 post-conception weeks, PCW), fetal hepatocytes (FH) 7-9 PCW (FH1) and 12-17 PCW (FH2), and adult hepatocytes (AH). (EPS 1.55 MB)

ESM 6 (PNG 350 KB)

High Resolution Image **Supplementary Figure 5.** **A.** Kaplan-Meier curves showing the differences in Disease-free interval (DFI) between the different EpiGT subgroups identified in the TCGA-LIHC cohort.** B.** Oncoplot summarizing the distribution of SNVs and INDELs mutational frequency in the TCGA-LIHC data set across the EpiGT subgroups for the top 20 mutated genes, left panel. Percentage of *TP53* and *CTNNB1* mutations in the different EpiGT subgroups. (EPS 1.71 MB)

ESM 7 (PNG 643 KB)

High Resolution Image **Supplementary Figure 6.** Heatmap representation of the five EpiG groups identified in the LIRI-JP dataset by unsupervised clustering using DAPC analysis, left panel. Elbow plot for optimal selection of the number of clusters, and scatter plot of unsupervised DAPC representing the centroid and ellipses of 95% confidence interval of the five EpiG subgroups, right panels. (EPS 3.18 MB)

ESM 8 (PNG 500 KB)

High Resolution Image **Supplementary Figure 7.** **A.** Kaplan-Meier curves showing the differences in overall survival between the different EpiGL subgroups identified in the LIRI-JP cohort. **B.** Distribution of tumor samples among different tumor stages, stages I - IV (left plot), or histological grades, G1-G4 (right plot), within the different EpiGL subgroups. **C.** Oncoplot summarizing the distribution of SNVs and INDELs mutational frequency in the LIRI-JP data set across the EpiGL subgroups for the top 20 mutated genes, left panel. Percentage of *TP53* and *CTNNB1* mutations in the different EpiGL subgroups. **D.** Distribution of tumor samples within each EpiGL subgroup according to the HCC subclassifications of Hoshida (S1, S2), Lee (A, B), Boyault (G1-G6), Chiang, and Roessler.** E.** Violin plots of the EpiG-HCC and HNF4a signatures analyzed using ssGSEA in the different EpiGL subgroups. (EPS 1.14 MB)

ESM 9 (PNG 320 KB)

High Resolution Image **Supplementary Figure 8. A. **HCC cell lines viability (fitness score) upon CRISPR/Cas9 drop-out screen for the indicated EpiGs. Negative values indicate reduced survival upon gene knockout. Data were retrieved from https://score.depmap.sanger.ac.uk/. **B.** Heatmap of the expression of epigenetic genes grouped in families and according to liver cancer cell lines transcriptomic subgroups (CL1, CL2, CL3) including the Cancer Cell Line Encyclopedia (CCLE). (EPS 1.03 MB)

ESM 10 (PNG 1.89 MB)

High Resolution Image **Supplementary Figure 9. **Heatmap of the expression of epigenetic genes grouped in families significatively downregulated (left panel) and upregulated (right panel) in non-responder patients to immunotherapy comparing with responder patiens in the GSE202069 dataset. (EPS 421 KB)

## Data Availability

No datasets were generated or analysed during the current study.

## Abbreviations

HCC: Hepatocellular carcinoma
ICIs: Immune checkpoint inhibitors
HBV: Hepatitis B Virus
HCV: Hepatitis C Virus
MASLD: Metabolic dysfunction-associated steatotic liver disease
TCGA: The Cancer Genome Atlas Research Network
PTM: Post-translational modification
ncRNA: Non-coding RNAs
EpiG: Epigenetic effector genes
SRA: Sequence read archive
TCGA-CDR: TCGA Pan-Cancer Clinical Data Resource
ICGC LIRI-JP: International Cancer Genome Consortium Liver Cancer Japanese Project
TMM: Trimmed mean of M-values
HB: Hepatoblast
PCW: Post-conception Weeks
FH: Fetal Hepatocyte
AH: Adult Hepatocyte
ssGSEA: Single sample gene set enrichment analysis algorithm
DAPC: Discriminant Analysis of Principal Components
PCs: Principal components
BIC: Bayesian Information Criterion
FDR: False Discovery Rate
MBGS: Mutated β-catenin Gene Signature
