# Supplementary figures and images for: Expression landscape of epigenetic genes in human hepatocellular carcinoma
Source: J Physiol Biochem. 2025 Jun 12;81(3):699–727. doi: 10.1007/s13105-025-01095-6 (PMC12373563; doi:10.1007/s13105-025-01095-6)

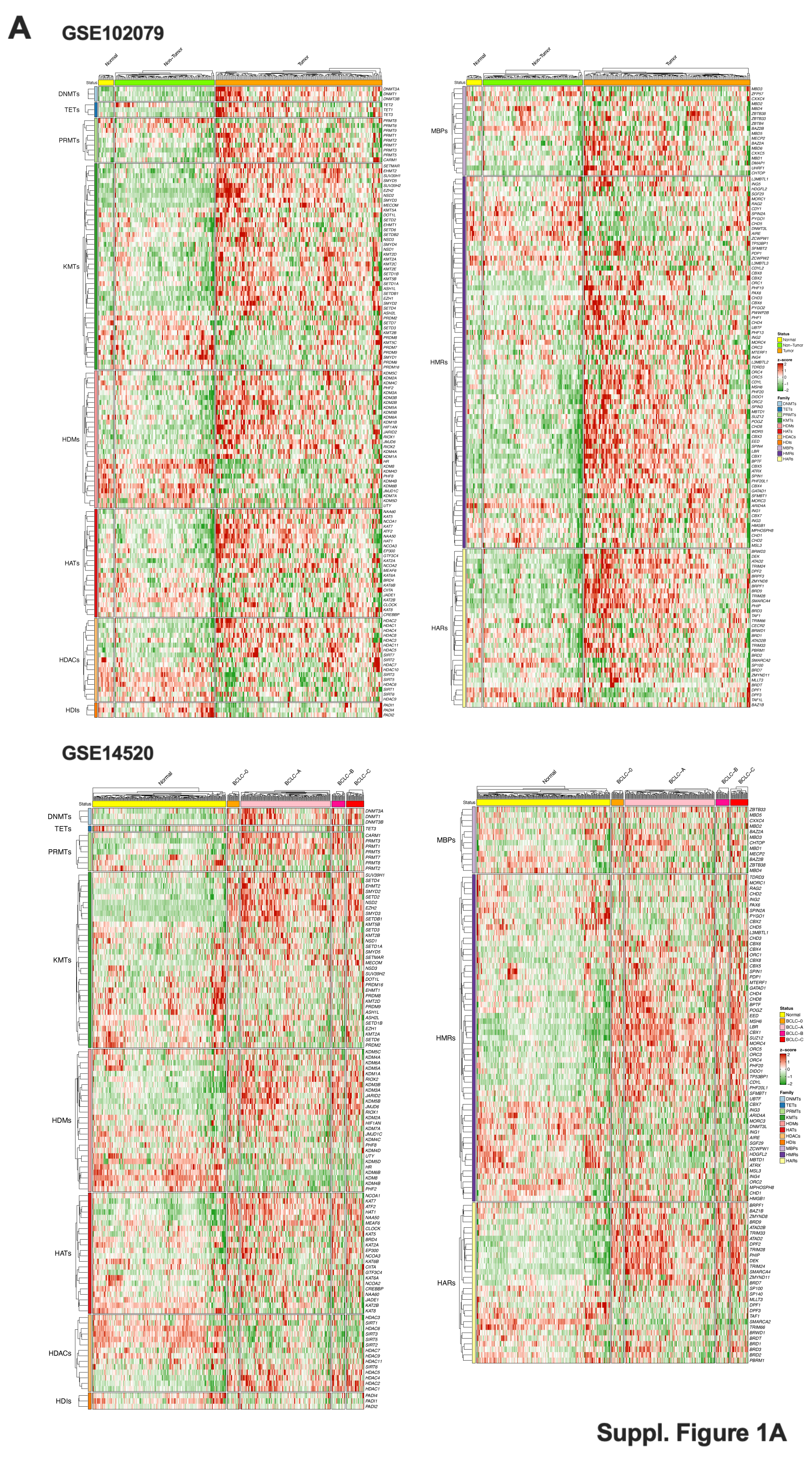

Supplement: Supplementary file 2 — (PNG 1.56 MB) [file 13105_2025_1095_Fig11_ESM.png]

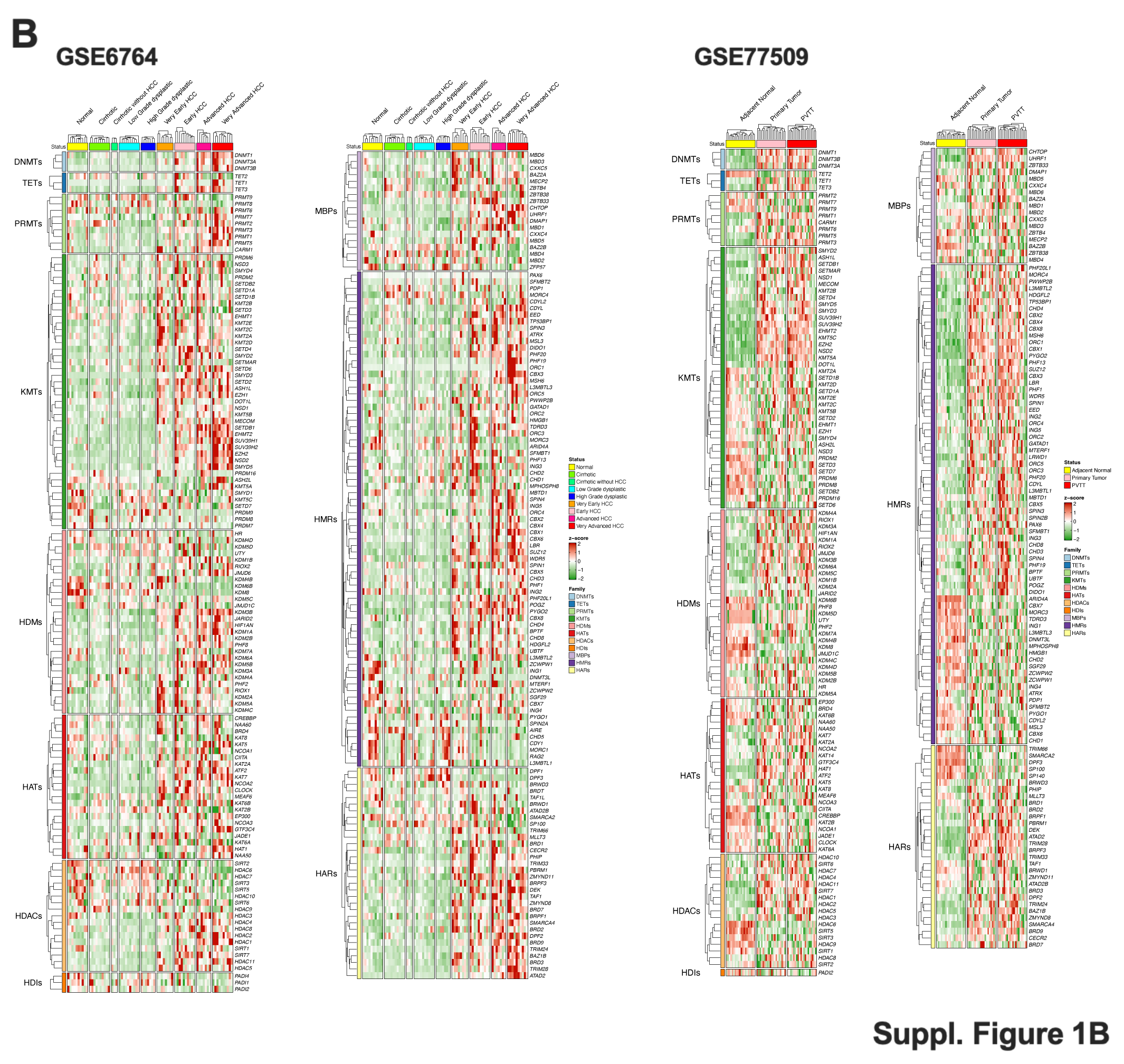

Supplement: Supplementary file 4 — (PNG 722 KB) [file 13105_2025_1095_Fig12_ESM.png]

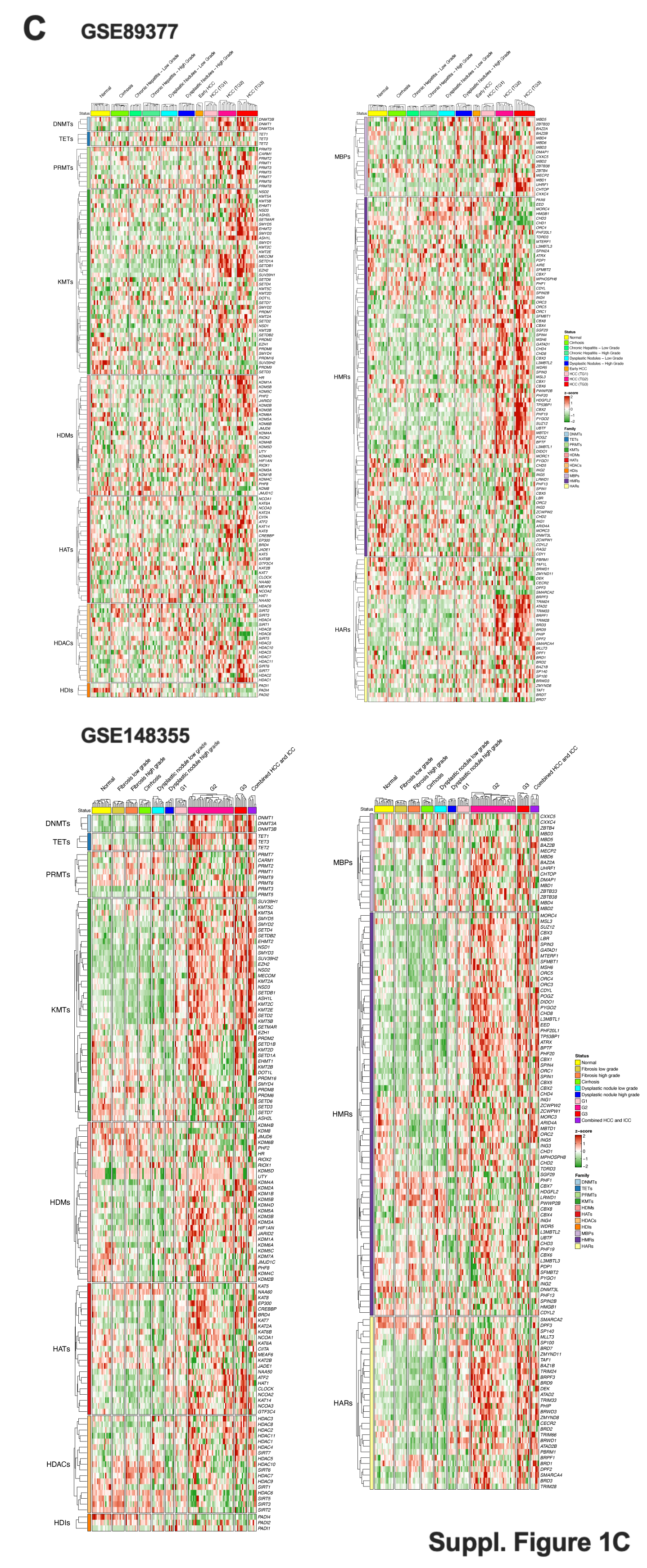

Supplement: Supplementary file 6 — (PNG 988 KB) [file 13105_2025_1095_Fig13_ESM.png]

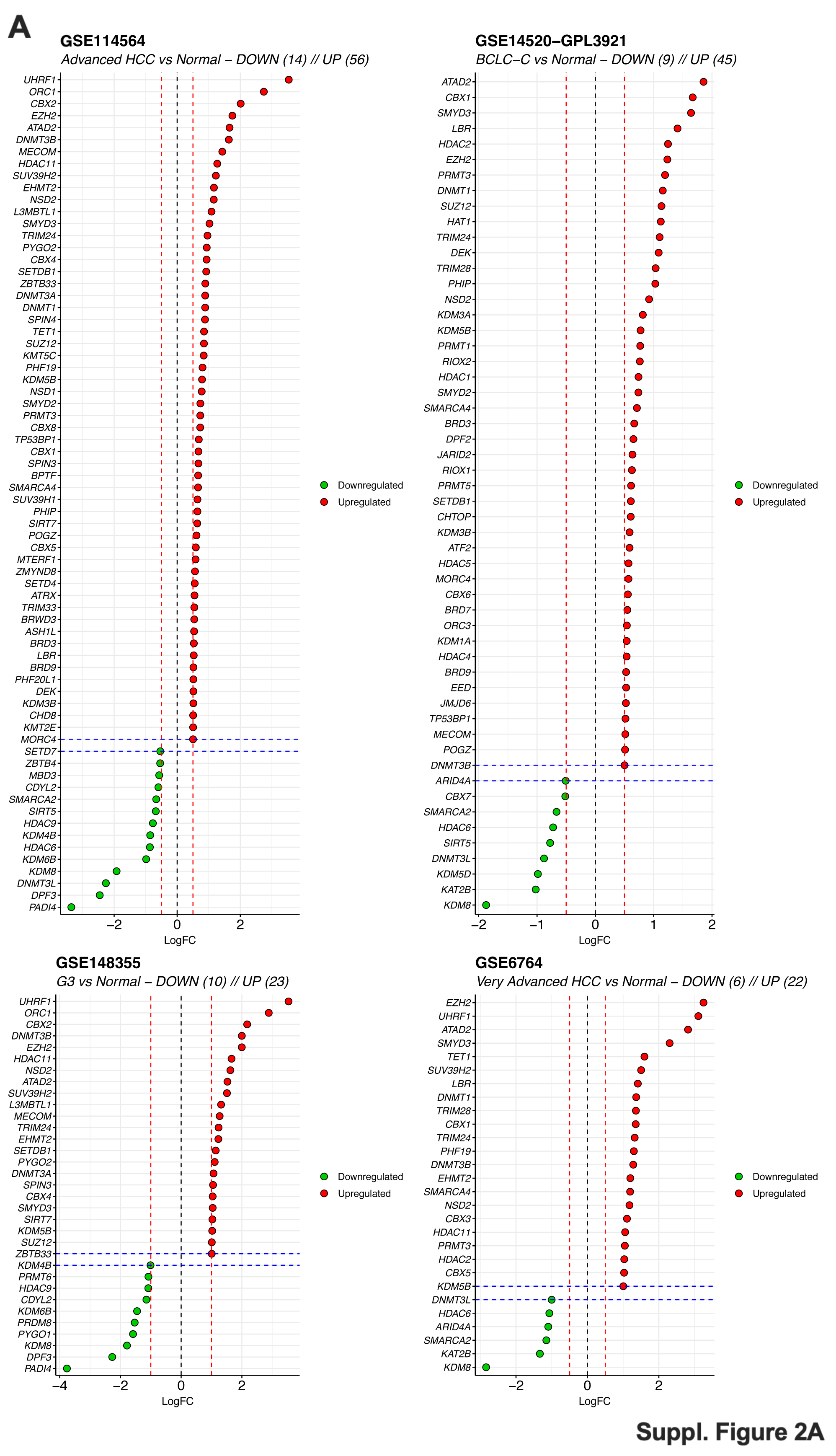

Supplement: Supplementary file 8 — (PNG 473 KB) [file 13105_2025_1095_Fig14_ESM.png]

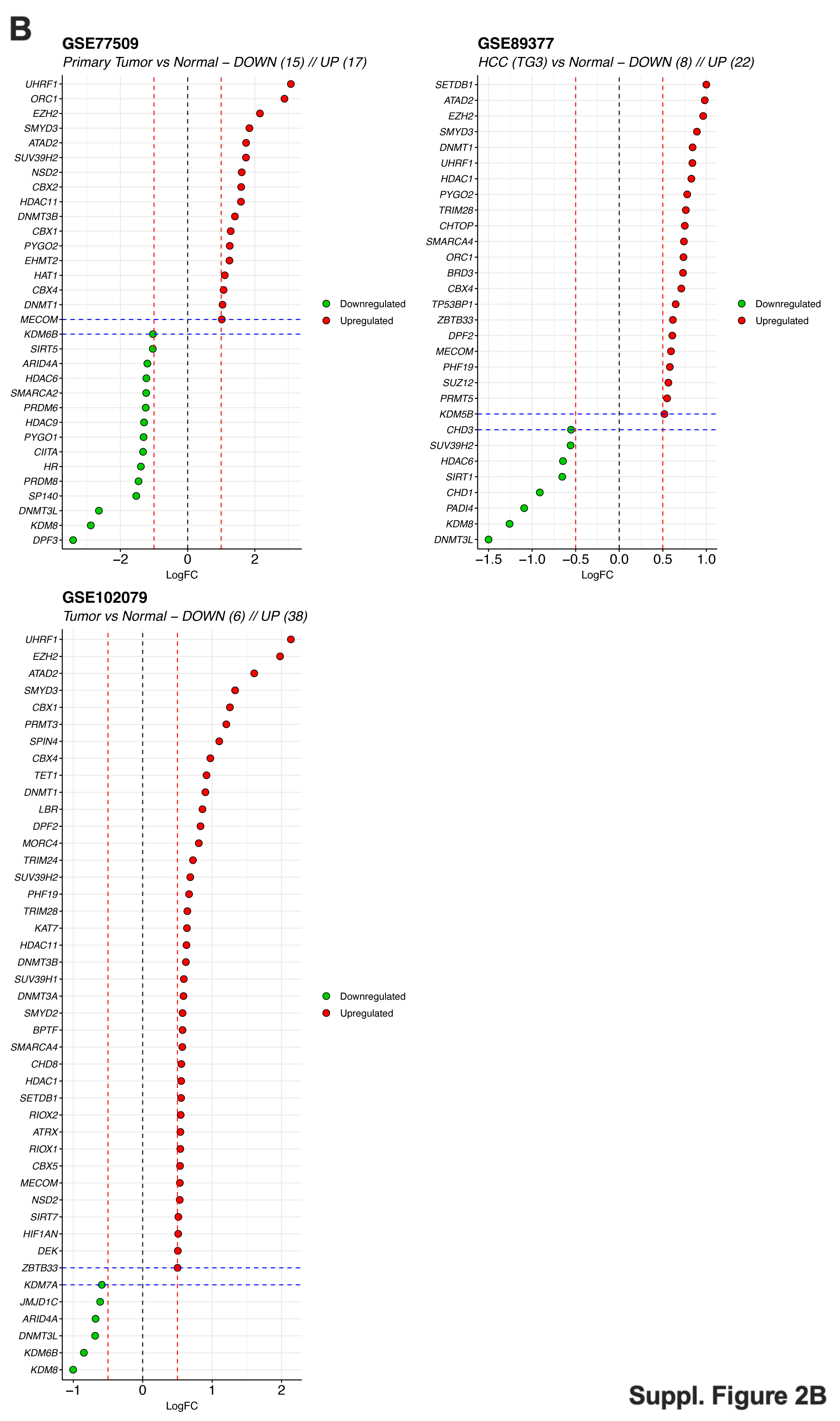

Supplement: Supplementary file 10 — (PNG 307 KB) [file 13105_2025_1095_Fig15_ESM.png]

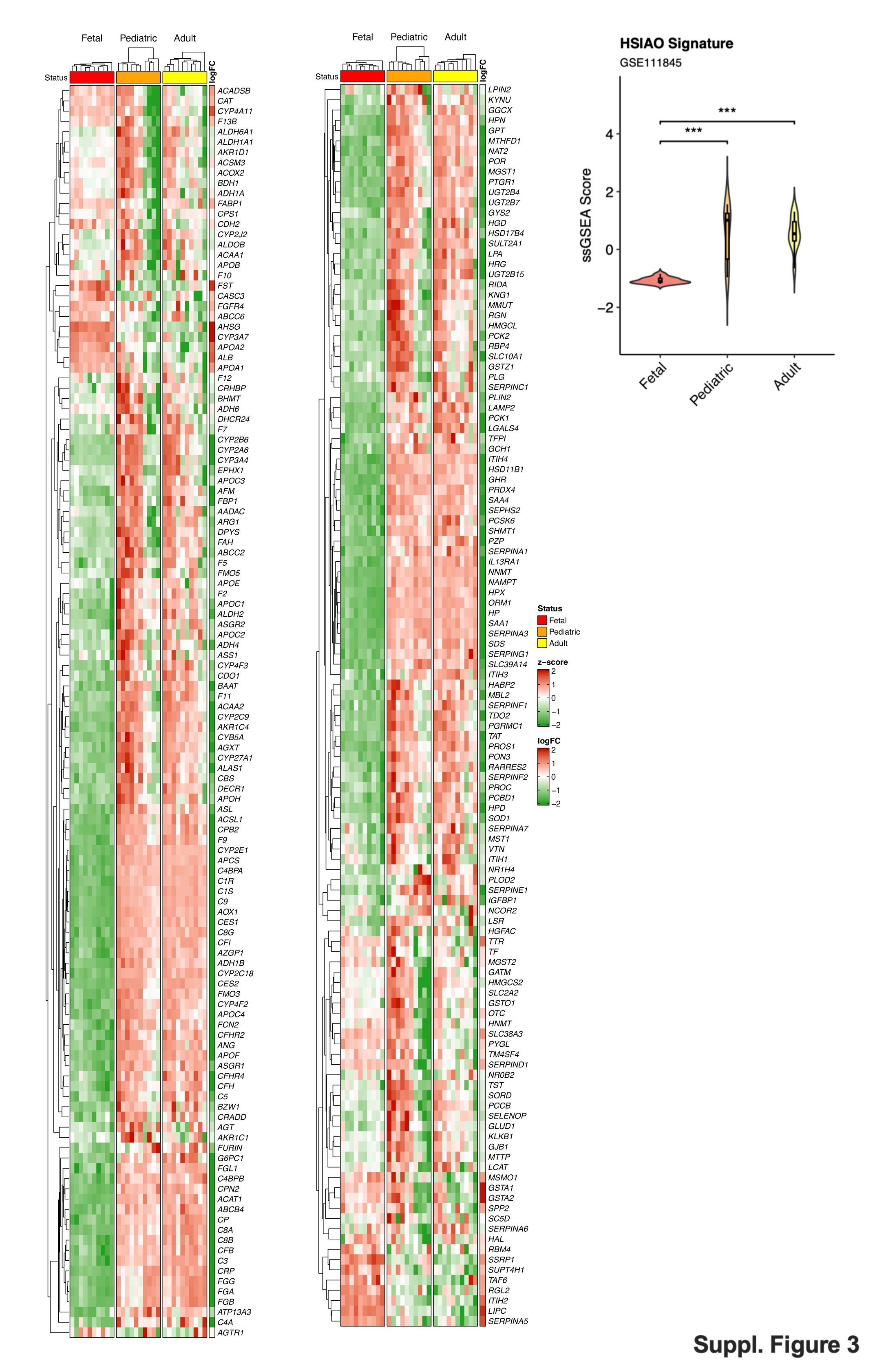

Supplement: Supplementary file 12 — (PNG 486 KB) [file 13105_2025_1095_Fig16_ESM.png]

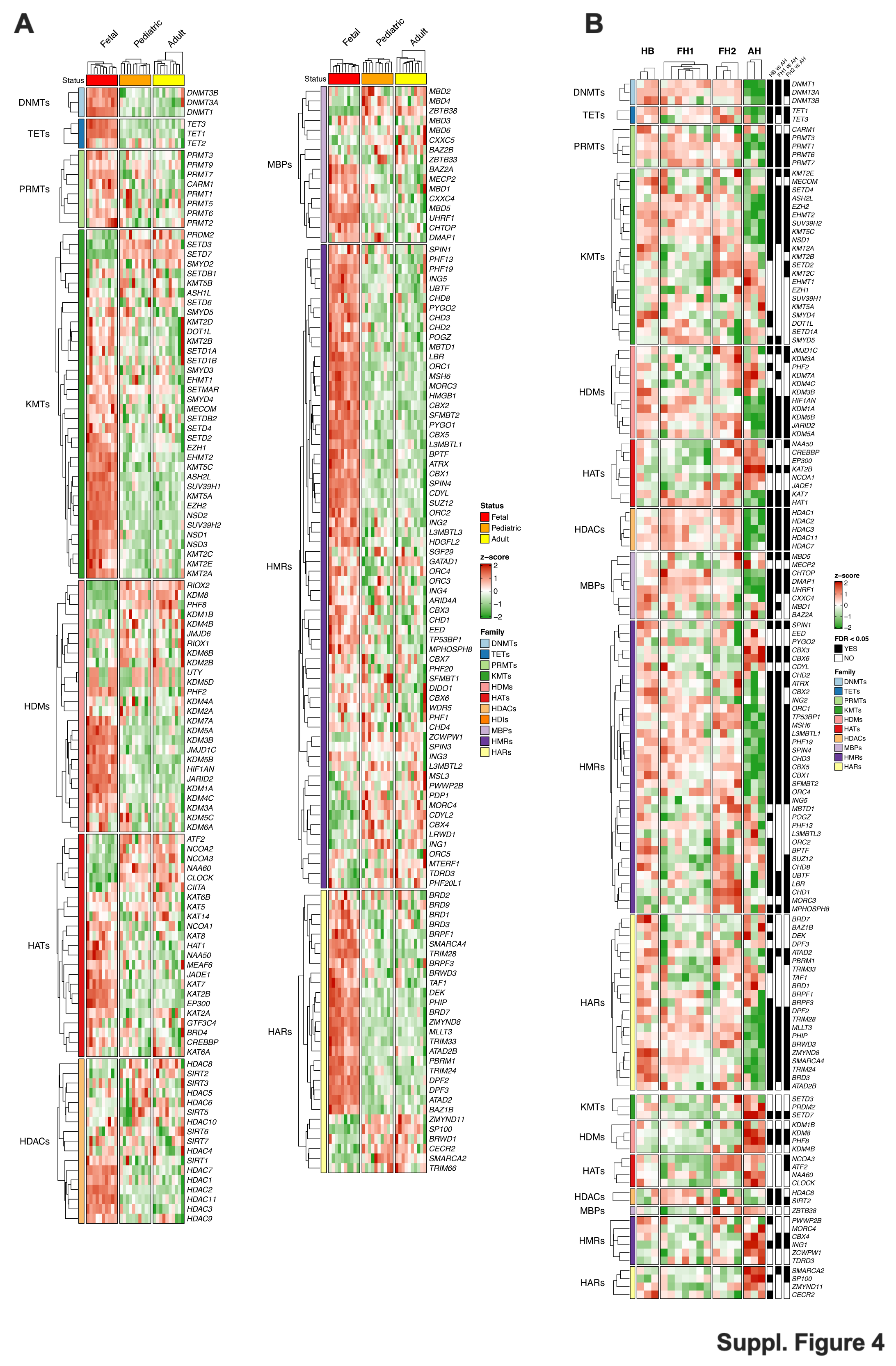

Supplement: Supplementary file 14 — (PNG 578 KB) [file 13105_2025_1095_Fig17_ESM.png]

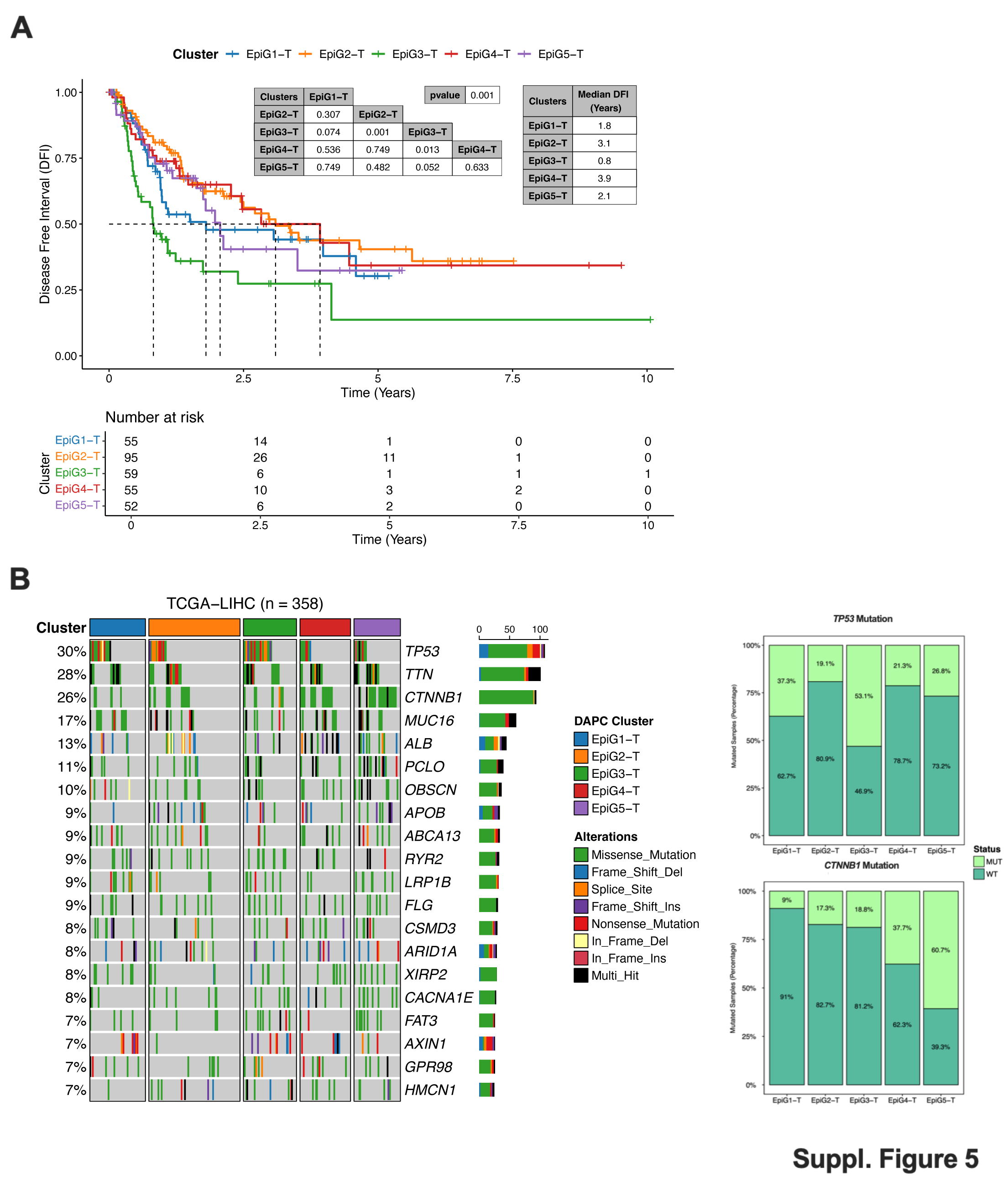

Supplement: Supplementary file 16 — (PNG 350 KB) [file 13105_2025_1095_Fig18_ESM.png]

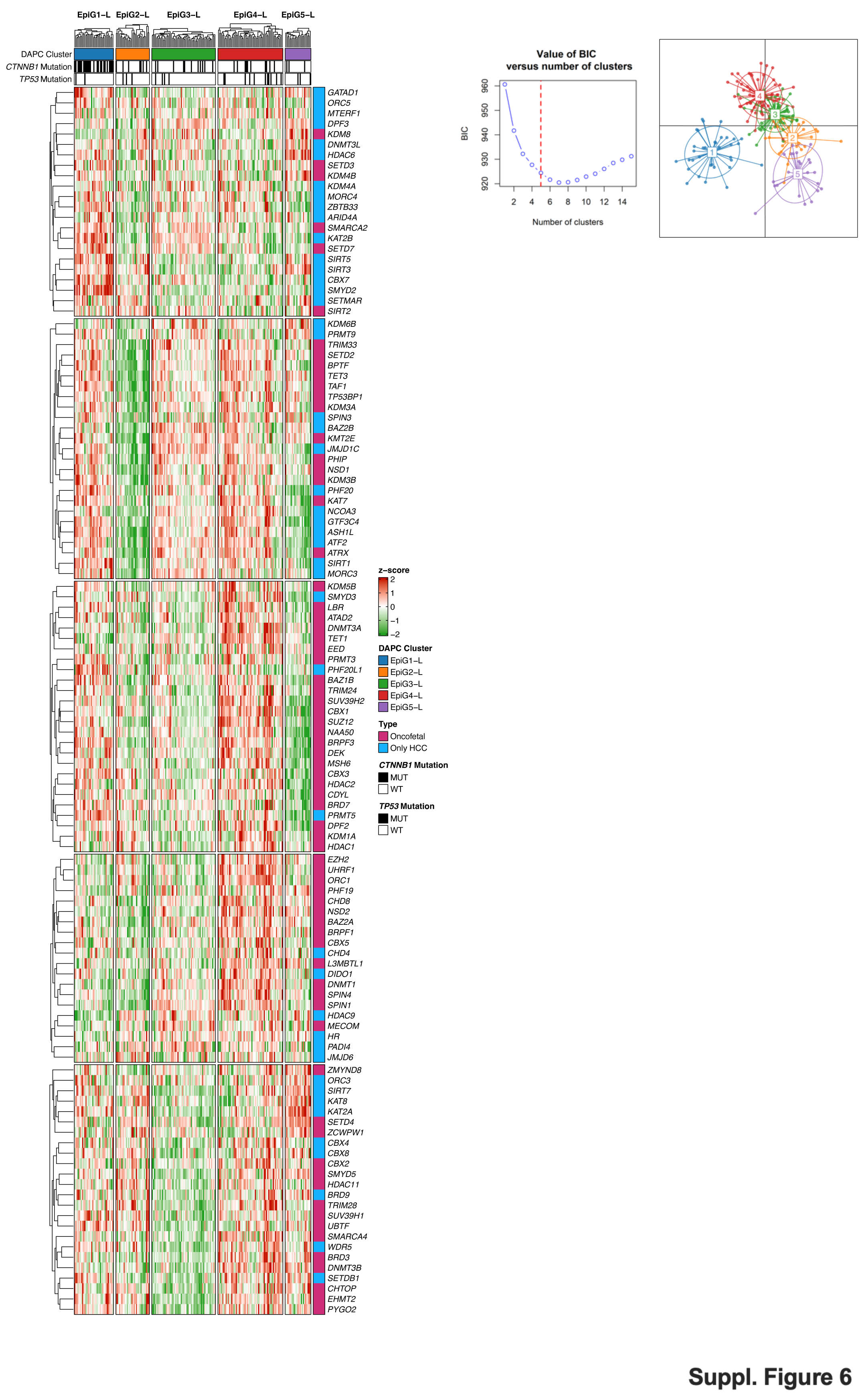

Supplement: Supplementary file 18 — (PNG 643 KB) [file 13105_2025_1095_Fig19_ESM.png]

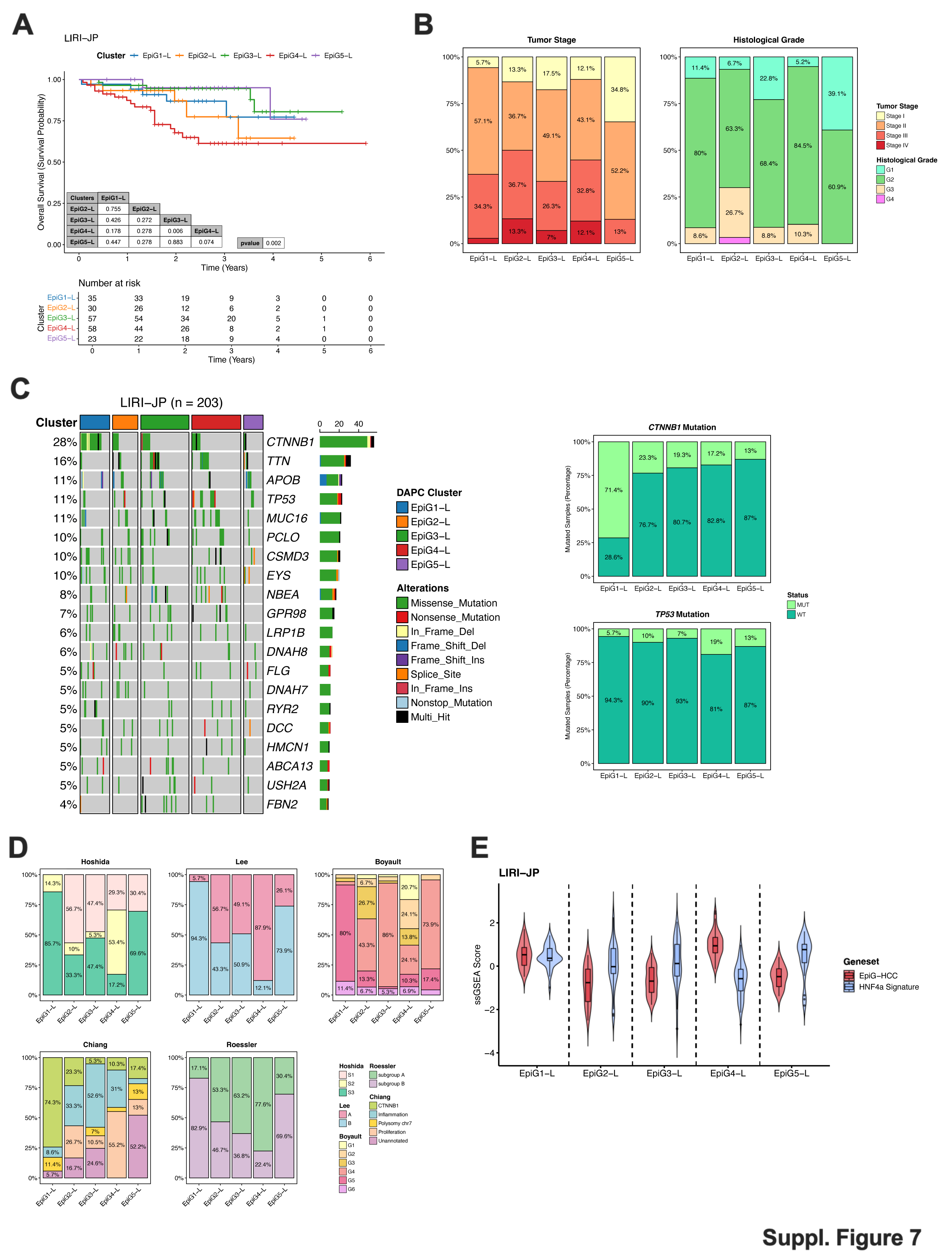

Supplement: Supplementary file 20 — (PNG 500 KB) [file 13105_2025_1095_Fig20_ESM.png]

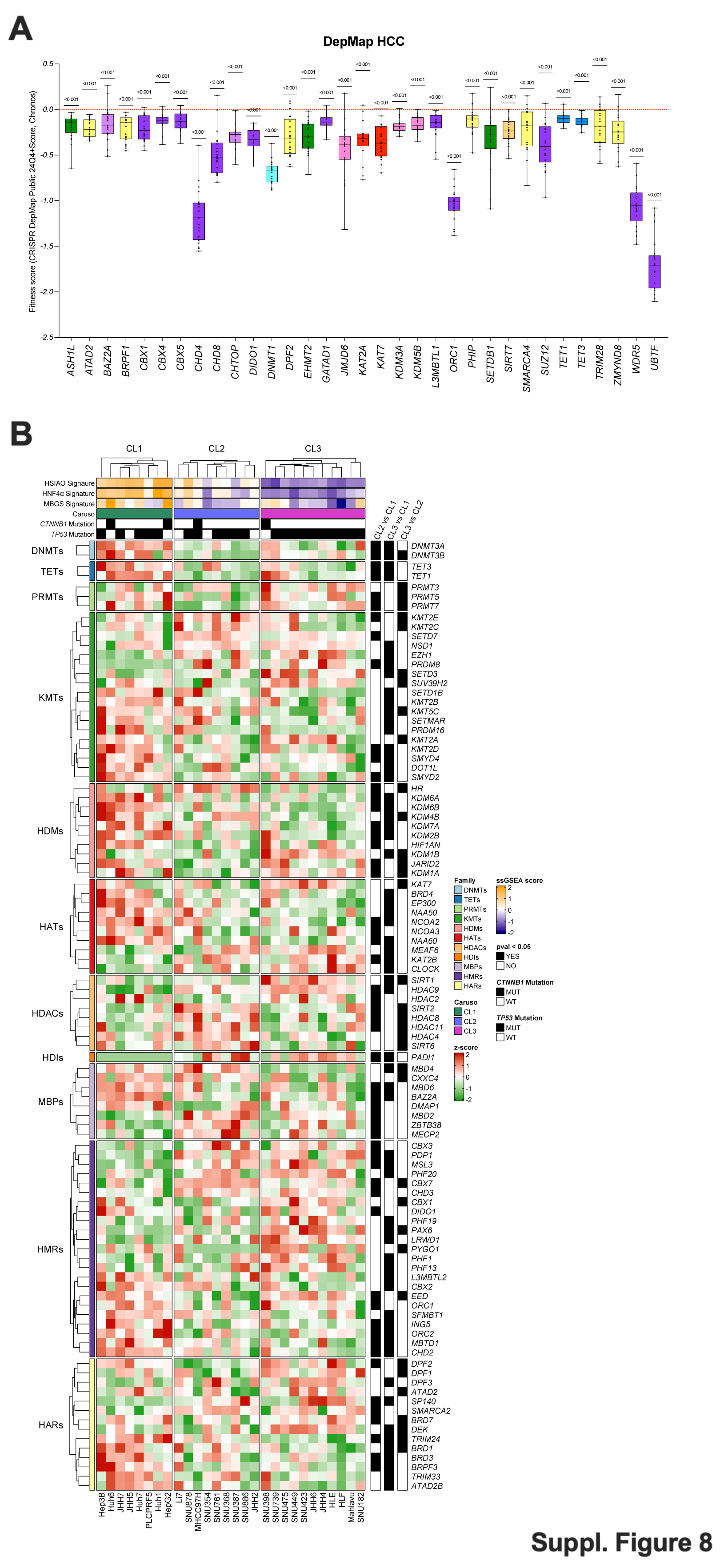

Supplement: Supplementary file 22 — (PNG 320 KB) [file 13105_2025_1095_Fig21_ESM.png]

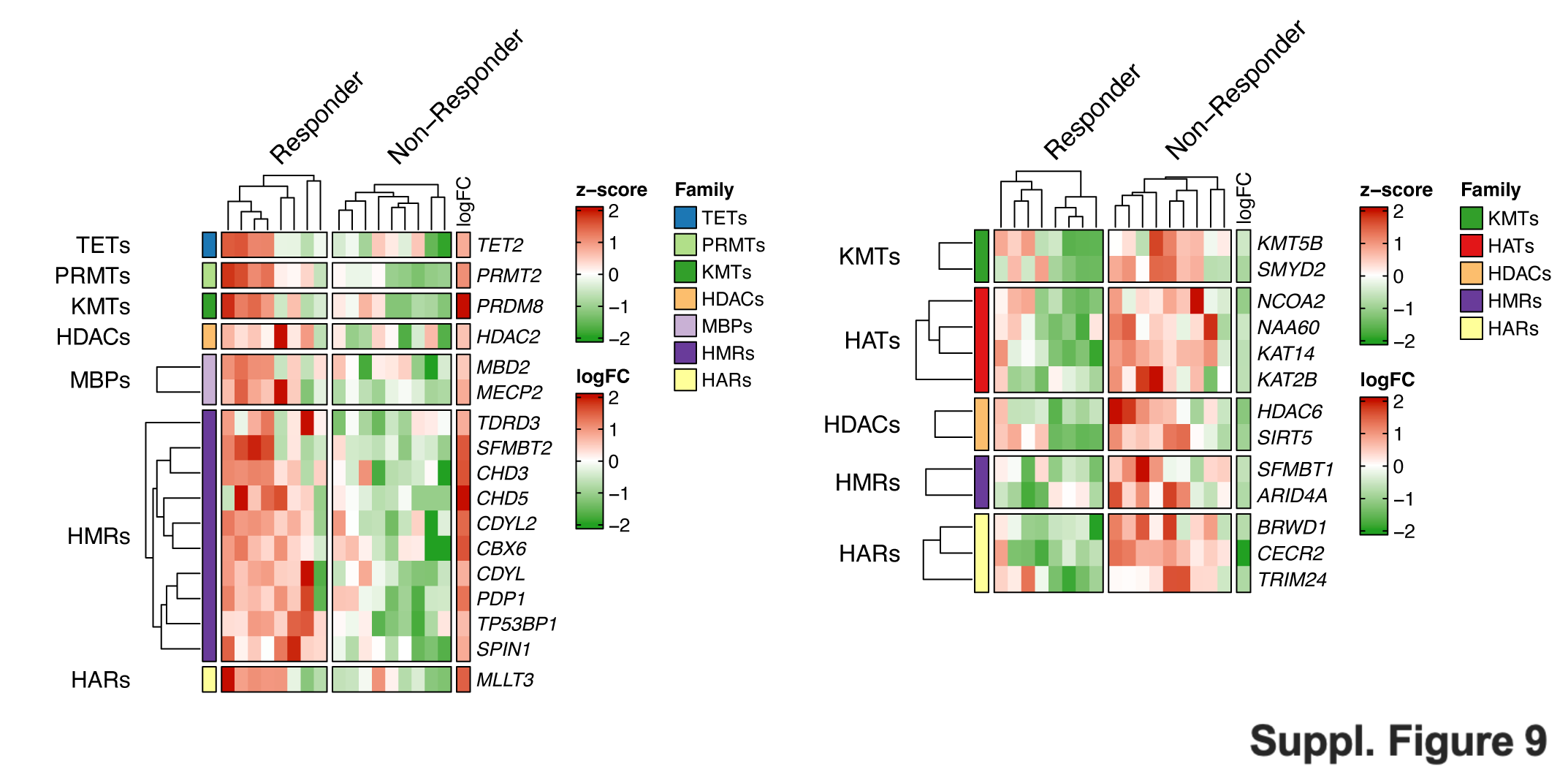

Supplement: Supplementary file 24 — (PNG 1.89 MB) [file 13105_2025_1095_Fig22_ESM.png]
